# Screening and vaccination as determined by the Social Ecological Model and the Theory of Triadic Influence: a systematic review

**DOI:** 10.1186/s12889-016-3802-6

**Published:** 2016-11-17

**Authors:** Anayawa Nyambe, Guido Van Hal, Jarl K. Kampen

**Affiliations:** 1 Faculty of Medicine and Health Sciences, University of Antwerp, Antwerp, Belgium; 2 Department of Epidemiology and Social Medicine, University of Antwerp, Antwerp, Belgium; 3 Biometris, Wageningen University, Wageningen, The Netherlands; 4 StatUA (Core Facility for Statistical Analysis), University of Antwerp, Antwerp, Belgium

**Keywords:** Screening, Vaccination, Multi-level ecological models, Social Ecological Model, Theory of Triadic Influence

## Abstract

**Background:**

Vaccination and screening are forms of primary and secondary prevention methods. These methods are recommended for controlling the spread of a vast number of diseases and conditions. To determine the most effective preventive methods to be used by a society, multi-level models have shown to be more effective than models that focus solely on individual level characteristics. The Social Ecological Model (SEM) and the Theory of Triadic Influence (TTI) are such models. The purpose of this systematic review was to identify main differences and similarities of SEM and TTI regarding screening and vaccination in order to prepare potentially successful prevention programs for practice.

**Methods:**

A systematic review was conducted. Separate literature searches were performed during January and February 2015 using Medline, Ovid, Proquest, PubMed, University of Antwerp Discovery Service and Web of Science, for articles that apply the SEM and TTI.

A Data Extraction Form with mostly closed-end questions was developed to assist with data extraction. Aggregate descriptive statistics were utilized to summarize the general characteristics of the SEM and TTI as documented in the scientific literature.

**Results:**

A total of 290 potentially relevant articles referencing the SEM were found. As for the TTI, a total of 131 potentially relevant articles were found. After strict evaluation for inclusion and exclusion criteria, 40 SEM studies and 46 TTI studies were included in the systematic review.

**Conclusions:**

The SEM and TTI are theoretical frameworks that share many theoretical concepts and are relevant for several types of health behaviors. However, they differ in the structure of the model, and in how the variables are thought to interact with each other, the TTI being a matrix while the SEM has a ring structure. The main difference consists of the division of the TTI into levels of causation (ultimate, distal and proximal) which are not considered within the levels of the SEM. It was further found that in the articles studied in this systematic review, both models are often considered effective, while the empirical basis of these (and other) conclusions reached by their authors is in many cases unclear or incompletely specified.

**Electronic supplementary material:**

The online version of this article (doi:10.1186/s12889-016-3802-6) contains supplementary material, which is available to authorized users.

## Background

Prevention refers to the efforts of society to promote, protect and sustain the health of the population. This paper focuses on vaccination and screening as primary and secondary prevention measures respectively. The aim of vaccination is to actively limit the incidence of disease by protecting the population from attack before being affected [1, 2], whereas screening tests identify asymptomatic individuals who may have the disease from those who probably do not [2].

All parts of the health system have an important role in the prevention of health problems [1]. Elder, Lytle, Sallis et al. [3] concluded that socio-ecological frameworks are essential in programs or studies that employ multi-level interventions and measurement strategies. Two key concepts of the ecological perspective help to identify intervention points for promoting health: 1) behavior both affects, and is affected by multiple levels of influence; 2) individual behavior both shapes, and is shaped by the social environment (causation) [4].

Accordingly, a number of multi-level models have been developed which incorporate all the different social and ecological factors that can affect health behavior in one single model, such as the Social Ecological Model and the Theory of Triadic Influence. Both provide a multilevel framework that can be used to study health behavior and the social environment.

### The Social Ecological Model

The SEM was developed out of the work of a number of eminent researchers including Bronfenbrenner’s Ecological Systems Theory (1979); McLeroy, Bibeau, Steckler et al. Ecological Model of Health Behaviors [5]; and Daniel Stokols’ Social Ecological Model of Health Promotion (1992, 2003) [4, 6].

The below systematic review considers all versions of the SEM. However, focus is on the SEM conceived by McLeroy, et al. [5], because it is one of the more common utilized versions. The SEM targets five levels/rings of influence for health related behaviors and conditions, which are: Intrapersonal (individual) factors for individual characteristics such as developmental history, knowledge, attitudes, behavior, self-concept and skills; Interpersonal processes (primary groups) these are social networks and support systems; Institutional (organizational) factors for social institutions with organizational characteristics and rules and regulations for operation; community factors for relationships among organizations, institutions and networks; and finally Public policy factors for local, state and national laws and policies [5]. The assumption is that people both influence and are influenced by those around them [4]. An additional file shows Figure S1: Social Ecological Model [see Additional file 1].

### The Theory of Triadic Influence

The TTI was developed by Flay, Snyder and Petraitis in 1994, as an integrative theory for health related behaviors [7]. It borrows from and builds of the ideas of Bronfenbrenner and Bandura [8]. The TTI assumes that the trail of a behavior is determined by one's decisions or intentions. It is organized in a 3 × 3 framework with three levels influence (causation) and three streams of influence [7].

The levels of causation include ultimate, distal, and proximal. Proximal or immediate variables are those that have direct effects on behavior and are under the control of an individual, although still influenced by the distal and ultimate factors. Distal level variables are divided into first level influences (social-personal nexus) and second level (evaluations and expectancies), and are composed of variables that individuals are likely to have some control over but not as much as proximal influences. Ultimate variables represent the underlying causes of behavior that are broad and relatively stable which individuals have little or no control over [7].

The types or streams of influence include: Intrapersonal influences which are characteristics that contribute to self-efficacy regarding specific behaviors; Interpersonal Social Influences which are the social situation/context or micro-environment that contribute to social normative beliefs about specific behaviors; and the Cultural-Environmental Influences which are multiple socio cultural macro-environmental factors that contribute to attitudes toward specific behaviors. Within each stream of influence are two sub streams, cognitive-rational and affective-emotional [7]. The TTI not only considers major influences of behavior as those within the three streams, but it also considers the interactions between stream paths and behavioral experience feedback loops. An additional file shows Figure S2: Theory of Triadic Influence [see Additional file 2].

### Relevance of a systematic review of SEM and TTI

The purpose of this systematic review was to identify the main differences and similarities of SEM and TTI regarding screening and vaccination in order to prepare potentially successful prevention programs for practice in general and cervical cancer prevention in Zambia in particular. By default, this systematic review benefits researchers who consider application of these models, by compiling the work of those authors who have had an opportunity to utilize them.

The SEM and TTI were selected for review because both models allow for the integration of multiple levels of influence to establish an overall view of health behavior change, in this case the uptake of vaccines and medical screening. Changing individual’s behavior by providing them with the necessary skills and motivation is only possible if environments and contexts are also considered. Both models target mechanisms of change at several different levels of influence involving both individual-level and environmental/policy-level interventions.

The SEM compared to TTI, is a more commonly utilized ecological model and has been applied for developing for example, screening interventions. For this reason the SEM is an appropriate model to discuss in this review. In contrast, the TTI was selected because compared to other multi-level models, it appears to greatly differ in structure and complexity with the SEM. Flay et al. [7], indicated that the TTI was used to conduct a screening study, suggesting this model is appropriate for studies focusing on screening and vaccination. The question is therefore justified, which of these models would be most effective in designing an effective screening and vaccination program.

Summarizing, this review addresses the research question: What are the main differences and similarities of the SEM and TTI regarding screening and vaccination programs?

## Methods

### Criteria for selecting studies for this review

The systematic review protocol was based on guidelines from the Cochrane Reviewer’s Handbook [9], to determine the extent that screening and vaccination as proposed by the SEM and TTI are different. The inclusion criteria for articles in the systematic review were the following:

- Geography - Include any country. Priority given to Sub-Saharan African countries.
- Time - Include studies starting from the year 2000 to date.
- Participants - Includes all people with health behavior affected from intrapersonal, interpersonal, organizational, community and policy level, in accordance with the SEM. For the TTI, from ultimate, distal and proximal levels; as well as intrapersonal, interpersonal social and socio-cultural environment streams.
- Disease - Priority is given to cancers and diseases that can be screened or vaccinated against. Also include substance abuse/risk behavior if they illustrate the use of the TTI.
- Exposure/Intervention - Primary and secondary prevention procedures including vaccination, screening methods and control of risk behavior.
- Comparison - May not be applicable in this study but will include people who do not practice primary and secondary prevention measures.
- Study Model - Include Social Ecological Model and Theory of Triadic Influence.
- Outcome - Set of optimal preventive measures by SEM and TTI.
- Language - Include only studies that are in English language.

All types of study designs were included in this review as long as they fulfilled the inclusion criteria. This is due to the fact that there are few studies that utilize the SEM and even less studies the TTI. Studies were considered eligible for the review if they involved human participants and followed the SEM or the TTI i.e. the full conceptual frameworks or modified versions of the frameworks.

The focus was on all diseases or conditions that can be vaccinated and or screened. Special interest was given to cancer because of future research plans. Substance abuse/risk behavior studies were included if the study illustrated the use of the TTI, regardless of whether there was a primary or secondary prevention intervention. This was due to the fact that very few studies that utilize the TTI address screening and or vaccination. The TTI was initially developed for substance abuse studies, therefore inclusion of studies that focus on substance abuse was relevant to illustrate the use of the TTI. The geographic location was not a factor in this review.

We also took into account the types of outcome measures, that is, the factors that predict health behavior choices such as knowledge, access to health care facilities and personal beliefs. The SEM and the TTI frameworks state that health behavior choices are influenced by a number of factors. When these factors are considered, they should therefore be able to predict whether a health behavior is practiced in this case, screening and vaccination. For the review, it was expanded to include the acquisition and cessation of risk behavior and substance abuse, as mentioned earlier, for the sake of the TTI which has relatively few studies that address screening and vaccination.

Literature searches were performed using Medline, Ovid, Proquest, PubMed, University of Antwerp Discovery Service and Web of Science. Searches were conducted during January and February of the year 2015. The searches were generated for a time span from 2000 to the present. Other criteria specified were English language, academic journal/articles and for databases where it was possible, human participants was specified.

The SEM produced a vast number of results compared to the TTI. It was therefore decided to narrow down the search for SEM studies to include only screening and vaccination, while for TTI, all studies were searched for due to the low number of results produced. Therefore, the search terms for the SEM studies were, Social Ecological Model AND Screening, Social Ecological Model AND Vaccination, Social Ecological Model AND Vaccine, Social Ecological Model AND Immunisation, Social Ecological Model AND immunization. The Boolean search term was the word 'AND'. For TTI studies, the search term was Theory of Triadic Influence. The full search strategies for each database are summarized in Table 1 below.

**Table 1:** Search terms and data bases searched

Databases searched	Limitations	Search terms
Medline	Date Published:2000/01/01–2015/12/31^a^ Language: English Document type: Academic Journals	Social Ecological Model AND^b^ Screening Social Ecological Model AND^b^ Vaccination Social Ecological Model AND^b^ Vaccine Social Ecological Model AND^b^ Immunisation Social Ecological Model AND^b^ Immunization Theory of Triadic Influence
Ovid	Date Published: 2000/01/01–2015/12/31^a^ Language: English Participants: Human	
Proquest	Date Published: 2000/01/01–2015/12/31^a^ Language: English Document type: Scholarly Journals	
PubMed	Date Published: 2000/01/01–2015/12/31 Language: English Participants: Human	
University of Antwerp Discovery Service	Date Published: 2000/01/01–2015/12/31 Language: English Document type: Academic Journals	
Web of Science	Date Published: 2000/01/01–2015/12/31^a^ Language: English Document type: Article	

^a^The range selected was between 2000 to present however, the database adjusts the dates when articles are available

^b^AND was the Boolean search term

### Data collection and analysis

Prior to commencing the review, a Data Extraction Form was developed to assist with data extraction. The form was divided into two sections. Only studies that had adequate inclusion criteria were fully evaluated by the Data Extraction Form. All studies had to have the first section completed and it had two parts:

i. General Information - This included date of data extraction, general publication information such as the title of the publication, the type of publication and source of the publication.
ii. Inclusion/Exclusion Criteria - These were questions based on the criteria for including studies such as indicating the study model that is used whether SEM or TTI, type of participants and setting, whether it was a primary or secondary prevention study, the presence of conclusions and finally the decision to include the study or not. If the study was not induced then the reason for exclusion would be stated.

If the article fulfilled the inclusion criteria then it will further be analyzed under the Characteristics of Included Studies section. This section was divided into seven parts:

i. General Study Details - For information on aims, research questions, hypothesis, study setting, units of observation, level of analysis, target disease and category of treatment investigated.
ii. Evaluation Design - Focused on indicating the type of study design, independent variables and the extent of use of the study model whether it is completely used or modified in some way.
iii. Data Sources 1 (Facilitators) - This was filled in only for studies that used facilitators to assist in obtaining data from participants. If more than one type of facilitator was used, then this part was repeated to cover all facilitators. It included information on source, sample size, sampling design and recruitment method.
iv. Data Sources 2 (Actual Participants) - This was information on actual participants of the study such as the target population, sample size, basic demographic characteristics, sampling strategy, data collection methods and finally main outcome measures that were assessed. This part was also repeated if more than one type of study participant was assessed in the study.
v. Analysis and Evaluation - This part focused on indicating the analysis plan be it for qualitative or quantitative study plans. If bias was noticed in the study it was indicated here.
vi. Results and Evaluation - This part illustrated the positive and negative outcome measures assessed the overview of the effectiveness of the SEM/TTI and whether effect size and power calculation was considered in the study.
vii. Other Information - Was a section to indicate whether ethical approval was obtained, if funding was available and whether references to other studies were given. A final question was to state whether further correspondence was necessary.

The form was pilot tested by two researches independently (JW & AN) to assess reliability and the final task of extracting the data was conducted by an independent researcher (AN). It was felt that having all this sections and parts was important to have a complete overview of the articles to be reviewed which were diverse in structure and content. An additional file has a copy of the complete Data Extraction Form [see Additional file 3].

In addition to systematically reviewing the articles for content matter, all articles passing the inclusion criteria were also screened for methodological consistency. This quality assessment addressed 7 issues vital for determining the empirical basis of the conclusions advanced in each article (see e.g., [10]), including the clarity of the research question(s), data collection methods, sampling plan, sample size, analysis method(s), conclusions, and limitations. Studies were not excluded from the review based on their quality rating. Quality assessment criteria can be found in Table 2.

**Table 2:** Data extraction form for quality assessment

Indicator	Categorization	Criteria
1. Clarity of CRQ(s)/hypothesis	0. Missing	At best, only sub-questions specified
	1. Unclear	CRQs supplied inappropriately (e.g., only in abstract) or incomprehensively (e.g., as identification of a research gap)
	2. Clear	
2. Clarity of data collection methods	0. Missing	None specified
	1. Unclear	Incompletely specified (e.g., type of interview/observation; application)
	2. Clear	
3. Clarity of sampling plan	0. Missing	None specified
	1. Unclear	Missing for at least one reported data collection method
	2. Clear	
4. Clarity of sampling size	0. Missing	None specified
	1. Unclear	Imprecise (e.g., ‘more than’), or missing for at least one reported data collection method
	2. Clear	
5. Clarity of analysis method	0. Missing	None specified
	1. Present	At least some description of data handling after collection (e.g., mention of transcription, CAQDA, grounded theory, content analysis, regression analysis, etc.)
6. Clarity of conclusions	0. Missing	None specified, or none with a relationship to research questions
	1. Present	At least one conclusion has a (however weak) link with one of the research questions
7. Clarity of limitations	0. Missing	None specified
	1. Unclear	Possible instrument effects and/or fallacies are mentioned but without further discussion
	2. Clear	research limitations are appropriately identified

The results of data extraction were input into a MS-Excel computer program. Basic descriptive statistics was utilized to summarize the general characteristics of the studies. This was facilitated with the use of IBM SPSS software. The main results review were identified and tabulated.

## Results

### Description of studies

Separate literature searches were conducted for the SEM studies and TTI studies. The literature searches yielded 57 potentially relevant articles in Medline, 172 titles in Ovid, 21 titles in Proquest, 56 titles in PubMed, 75 titles in University of Antwerp Discovery Service and 58 titles in Web of Science for SEM studies. A total of 439 articles, and without duplicates 290 potentially relevant articles were found.

As for the TTI studies, literature searches yielded 18 titles in Medline, 54 titles in Ovid, 23 titles in Proquest, 22 titles in PubMed, 65 titles in University of Antwerp Discovery Service and 46 titles in Web of Science. In a brief systematic review on the TTI, it was discovered that a study was conducted on colorectal cancer screening using this theory. Therefore Google Scholar was used specifically to find this study because of its relevance for this research. A total of 229 articles, and without duplicates 131 potentially relevant articles were found.

**Fig. 1:**
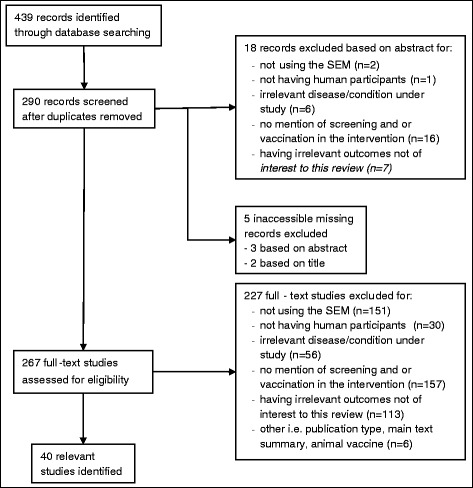
Prisma study flow diagram of search results for SEM. Note: Some studies were excluded for more than one reason. For studies excluded based on abstract, 9 studies were excluded for 1 reason; 6 studies excluded for 2 reasons; 2 studies excluded for 3 reasons; and 1 study excluded for 4 reasons. For studies excluded based on full-text, 71 studies were excluded for 1 reason; 73 studies excluded for 2 reasons; 48 studies excluded for 3 reasons; 22 studies excluded for 4 reasons; and 13 studies excluded for 5 reasons

**Fig. 2:**
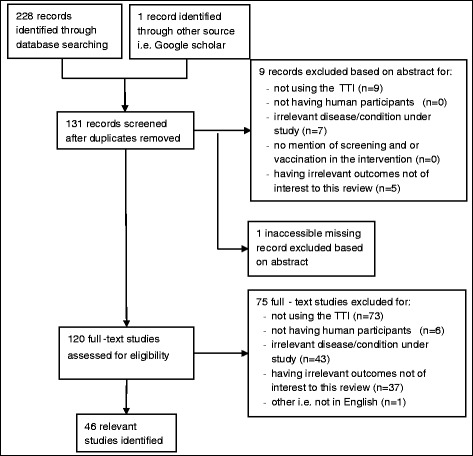
Prisma study flow diagram of search results for TTI. Note: Some studies were excluded for more than one reason. For studies excluded based on abstract, 1 study was excluded for 1 reason; 4 studies excluded for 2 reasons; and 4 studies excluded for 3 reasons. For studies excluded based on full-text, 30 studies were excluded for 1 reason; 11 studies excluded for 2 reasons; 28 studies excluded for 3 reasons; and 6 studies excluded for 4 reasons

Based on the number of potentially relevant articles, it was decided to find and evaluate all articles with the inclusion and exclusion criteria. Articles that could not be easily obtained were evaluated based on abstract and title. After strict evaluation of the articles 40 SEM studies and 46 TTI studies were included. A summary of these results are found below as study flow Figs. 1 and 2.

There were five main reasons for studies not to be included in review. These reasons include not applying the SEM or the TTI in the research, having non-human participants, addressing an irrelevant disease or condition, for SEM studies specifically not addressing screening or vaccination, and finally having irrelevant outcomes. Most studies that were eliminated had a combination of these reasons. Studies that were eliminated for only a single reason were for either not applying the models in the research or not having human participants.

There were a total of 251 possible SEM studies excluded. Majority were excluded for not being a study that addresses screening or vaccination (*n* = 173), this is followed by studies were found but did not apply the SEM in the research (*n* = 153). As for the possible articles involving the TTI, a total of 85 studies were excluded. Main reason being that the study did not apply the TTI in the research (*n* = 82) and this is followed by assessing an irrelevant disease or condition in the study (*n* = 50).

Another six studies (SEM *n* = 5; TTI *n* = 1) were not included in the review because of inaccessibility of the publication and are therefore still awaiting classification. The brief information provided by the titles and/or abstracts of these articles makes exclusion inconclusive. Efforts have been made to access the full text from the authors of these articles. However, there has not been any response up to the date of submission of this systematic review. An additional file lists these studies in Table S1 [see Additional file 4].

The Data Extraction Form used closed questions that focused on items that would be of interest to the review. Table 3 below summarizes the top three most common items from the result fields in the articles based on frequency. The frequency of the results below the top three items is relatively low. The Data Extraction Form allowed for an option of “other” for items that did not fall under the predetermined items of interest. This table excludes the result “other” even if it had been in high frequency because it is composed of a combination of random items. An additional file shows the complete compilation of results in Tables S2 and S3 [see Additional file 4].

**Table 3:** Summary of most common results

Field/Topic	Item(s)	Articles using SEM		Articles using TTI	
		Frequency	Percent	Frequency	Percent
Location	USA	25	62.5	17	37.0
	Canada	2	5.0	1	2.2
	India	2	5.0	-	0.0
	Netherlands	-	0.0	7	15.2
	Australia	1	2.5	3	6.5
Study participants^a^	Women	14	26.9	1	1.6
	Books/journal articles	10	19.2	4	6.3
	Men	7	13.5	-	0.0
	Students	-	0.0	22	34.9
	Parents/guardians	1	1.9	9	14.3
	Adolescents	-	0.0	8	12.7
Aim/Objective	To form or evaluate interventions	12	30.0	14	30.4
	Determine the acceptance or non-acceptance of screening, vaccination or treatment	11	27.5	1	2.2
	Determine the cause of behavior	7	17.5	19	41.3
	Explore views	3	7.5	2	4.3
Disease/Condition	Breast cancer	6	15	-	0.0
	Colorectal cancer	4	10	1	2.2
	Cervical cancer	3	7.5	-	0.0
	Substance abuse	-	0.0	23	50.0
	Skin cancer	-	0.0	1	2.2
Intervention	Screening	29	72.5	2	4.3
	Vaccination	9	22.5	-	0.0
	Substance Abuse/Risk Behavior	5	12.5	20	43.5
Study design (primary)	Cross-sectional	5	12.5	16	34.8
	Case study	4	10.0	1	2.2
	Cohort	2	5.0	4	8.7
	Longitudinal	0	0.0	9	19.6
	Randomized controlled trial	0	0.0	5	10.9
Study design (secondary)	Simple overviews	12	30.0	4	8.7
	Systematic reviews	3	7.5	2	4.3
	Guideline	1	2.5	3	6.5
Sampling strategy^a^	Judgmental	14	26.9	17	27.0
	Convenience	11	21.2	20	31.7
	Simple random	4	7.7	9	14.3
	Stratified	4	7.7	3	4.8
	Snowball	1	1.9	8	12.7
	Not reported	11	21.2	19	30.2
Data collection method^a^	Secondary data	15	28.8	12	19.0
	Interviews	14	26.9	9	14.3
	Questionnaires	13	25.0	41	65.1
	Not reported	1	1.9	1	1.6
Outcome variables^a^	Screening was practiced	19	36.5	2	3.2
	Screening is not practiced	3	5.8	-	0.0
	Vaccination is practiced	8	15.4	-	0.0
	Vaccination not practiced	5	9.6	-	0.0
	Risk behavior is practiced	1	1.9	40	63.5
	Risk behavior is not practiced	2	3.8	19	30.6
	Not reported	8	15.4	6	9.5
Positive predictors	Positive influences and surroundings	18	45.0	21	45.7
	Having knowledge or awareness	18	45.0	5	10.9
	Recommendations from healthcare providers	13	32.5	1	2.2
	Access to healthcare providers or facilities	13	32.5	3	6.5
	Personal beliefs	6	15.0	13	28.3
	enforcing policies/rules	9	22.5	6	13.0
	Not reported	10	25.0	13	28.3
Negative predictors	Negative personal beliefs	15	37.5	17	37.0
	Negative influences and surroundings	13	32.5	28	60.9
	Lack of access to healthcare providers or facilities	11	27.5	2	4.3
	Culture of the group of people	4	10.0	7	15.2
	Not reported	15	37.5	11	23.9

^a^For these items the percentage is calculated over 52 for the SEM and over 63 for the TTI due to multiple study participants. The percentage in general is calculated over the total number of accepted studies, which are 40 for the SEM and 46 for the TTI

As seen from the table most studies for both SEM and TTI, were undertaken in the USA. However, it should be noted that some studies took place in multiple countries (SEM *n* = 4; TTI *n* = 3). When it came to study participants, some studies assessed more than one type of participant. For this review, sampling strategy, data collection method and outcome measures for each unique participant group were considered. However, some this information especially from the additional study participants in both SEM and TTI studies was not as emphasized as the primary participant data resulting into some cases of missing or not reported data. In articles using the SEM, nine studies had more than one type of participant group. The reported sample sizes ranged from 1–70,121. In TTI studies, a total of ten studies had more than one type of participant group. Reported sample size ranged from 10–36,000.

Considering disease or condition under study, the Data Extraction Form focused on different types of cancers, cancer in general, substance abuse and then grouped all other diseases and conditions under the option "other". This was because cancer in particular cervical cancer is a disease of interest for future study and for the TTI substance abuse is the most common condition researched. In that regard, for the SEM, cancer in general was studied in 4 studies (7.7%) and this included studies that assessed both breast and cervical cancer. 22 studies (42.3%), focused on other diseases or conditions such as diabetes, HIV AIDS and Obesity. As for TTI studies, 21 studies (33.3%), focused on other diseases or conditions such as obesity, risk behavior and HIV AIDS.

The outcomes were divided into positive and negative predictors. Positive predictors were factors that would cause someone to go for screening, vaccination or to practice non-risk behavior. Negative predictors were the opposite of these. These factors were variable depending on the focus or nature of the study.

Finally, not all accepted studies using the SEM and the TTI reported on the overall effectiveness of these models. For studies that used the SEM, 17 studies (42.5%) reported on effectiveness of the model and they all considered the model to be efficient. From the studies that gave an overview of the effectiveness of the TTI, ten studies reported the TTI to be effective (21.7%), while seven reported it to be effective if modified (15.2%).

### Quality Assessment

The studies were not checked for risk of bias in measurement because there was insufficient detail provided in all of the papers. Therefore, quality assessment was conducted in accordance to the data extraction form for quality assessment which allows for maximum amount of twelve points (see Table 2 for the scoring system). Table 4 below summarizes the overall quality of the SEM and TTI studies. In general, the mean overall result for studies using the SEM is 7.4 and for TTI studies 9.3. It is apparent that studies utilizing the TTI were of slightly better quality compared to studies using the SEM. This may be due to the fact that more of the articles using the SEM were secondary simple overview articles and therefore did not elaborate much on methodology and study design compared to articles using the TTI. For both SEM and TTI, the majority of missing data is in the reporting of the sampling plan, analysis method and limitations. On the other hand, all studies reported conclusions to their research. These conclusions are trusted to give a comparison of the SEM and TTI, but it should be noted that the empirical basis on which authors based their conclusions remains unclear in most studies. An additional file shows Tables S4 and S5 which have a complete view of quality assessment of studies using the SEM and TTI respectively [see Additional file 4].

**Table 4:** Summary results of the data extraction form for quality assessment

Indicator	Categorization	Articles using SEM		Articles using TTI	
		Frequency	Percent^a^	Frequency	Percent^a^
1. Clarity of CRQ(s)/hypothesis	0. Missing	10	25.0	6	13.0
	1. Unclear	19	47.5	18	39.1
	2. Clear	11	27.5	22	47.8
2. Clarity of data collection methods	0. Missing	10	25.0	5	10.9
	1. Unclear	3	7.5	1	2.2
	2. Clear	27	67.5	40	87.0
3. Clarity of sampling plan	0. Missing	17	42.5	17	37.0
	1.Unclear	4	10.0	2	4.3
	2. Clear	19	47.5	27	58.7
4. Clarity of sampling size	0. Missing	13	32.5	7	15.2
	1. Unclear	3	7.5	8	17.4
	2. Clear	24	60.0	31	67.4
5. Clarity of analysis method	0. Missing	17	42.5	8	17.4
	1. Present	23	57.5	38	82.6
6. Clarity of conclusions	0. Missing	-	0.0	-	0.0
	1. Present	40	100.0	46	100.0
7. Clarity of limitations	0. Missing	16	40.0	8	17.4
	1. Unclear	6	15.0	2	4.3
	2. Clear	18	45.0	36	78.3

^a^The percentage is calculated over the total number of accepted studies, which are 40 for the SEM and 46 for the TTI

## Discussion

### Characteristics of SEM

The SEM has been applied in a number of prevention method studies focusing on breast, colorectal and cervical cancers. The majority of the studies utilized the SEM developed by McLeroy et al. (2008). However, it should also be noted that, some studies modified the model by addressing only specific independent variables or integrating other models that were of interest to the researchers. Furthermore, the studies either involved a single or several types of study participants. Whereas, the researchers would either observe a single group and investigate their views in regards to the different constructs of the model, or they would observe the interaction between groups of participants.

In regards to study aims, most SEM studies looked at evaluating interventions followed by accessing the acceptance or non-acceptance of a preventive measure, for instance. In terms of outcomes, positive experiences lead to positive outcomes, while negative experiences lead to negative outcomes, as expected.

In practice, the SEM is advocated to be an effective model in determining vaccination and screening behavior. Perhaps this is due to the flexibility of the SEM in regards to individual variables within the levels. For instance, a study by Maar, Wakewich, Wood et al. [11] applied the SEM to increase screening participation and concluded that cervical screening promotion needs to be implemented at multiple, culturally compatible levels. In regards to vaccination, a study by Kumar, Quinn, Kim et al. [12] validated all levels of the SEM as determinants of vaccine uptake. Furthermore, according to another study, the SEM was said to provide a useful schematic to assess how systems facilitate or create barriers to vaccinate; how individual level factors and community discourse, beliefs, and practices shape a person's perceptions and decisions to vaccinate [13].

### Characteristics of TTI

Studies that used the TTI mainly focused on substance abuse and risk behavior. This is not a surprise as the genesis of the TTI occurred after a careful review of the substance use literature [7]. It should be noted that all studies used the TTI as developed by Flay et al., however, some authors modified the model to only assess variables of interest.

In regards to study aims, the TTI generally aims to determine the cause of behavior. In this case, what would cause someone to practice a preventive measure? Most studies were cross-sectional in nature. However, it was expressed that a longitudinal study may be more effective because a follow up determining whether the population of interest will practice the risk behavior is beneficial.

Studies either involved a single or several types of study participants in relation to constructs of the model. For instance, a study may primarily focus on adolescents and then might also question parents to identify if adolescents with parents who practice a certain behavior will emulate them. In terms of outcomes, as with the SEM, positive experiences lead to positive outcomes and vice versa, which is expected.

In practice, the TTI is advocated to effectively predict substance abuse and risk behavior. However, the TTI might prove to be too complex for screening or vaccination decision studies. As evident in the study by Kremers, Mesters, Pladdet et al. [14], that applied the TTI to determine colorectal cancer screening participation and non-participation decisions. The model proved to be useful in explaining screening participation behavior, but it was recommended to redefine the operationalization of some variables to improve reliability. Nevertheless, the only other study that focused on screening for a HIV AIDS intervention [15], did not specify the effectiveness of the TTI. None of the studies found by this systematic review focused on vaccination.

Other studies that specified that the TTI is better modified, found it was too complex for guidance in the field [16], complexity in analysis of the relationship between cultural and social context [17], cultural stream factors were of less significance and the levels of influence were not hierarchical [18], and further studies stated the lack of data to properly operationalize the model thus reducing its effectiveness [19, 20].

### Similarities and Differences of SEM and TTI

The SEM and TTI are valuable theoretical frameworks that are relevant for several types of health behaviors. Generally, these frameworks share similar theoretical concepts. In terms of composition, the streams of influence of the TTI correspond with the levels of the SEM. Whereas, the TTI intrapersonal stream would be equated to the SEM intrapersonal level, the TTI social situation stream to the SEM interpersonal level and finally the TTI cultural environmental stream to a combination of the SEM organizational, community and policy levels.

Basically, these theoretical frameworks differ in structure of the model and how the variables interact with each other. The TTI being a matrix while the SEM having a ring or level by level structure. In spite of most of the articles for both SEM and TTI, being from the USA, the basic function of these models has been expressed. However, depending on the society the model is being used in, some of the concepts and interactions may need to be modified to fit with the local situation. For instance, while it is common to have family doctors in the USA which places healthcare providers in a more interpersonal level, in third world countries this is not necessarily the norm and healthcare providers may be positioned at a different level of interaction.

The main difference consists of the division of the TTI into levels of causation (ultimate, distal and proximal) which are not considered within the levels of the SEM. The TTI separates levels of causation from ecological domains by making them independent dimensions within each ecological domain. This finding is consistent to the description by Flay et al. [7], who also stated that the TTI overcomes problems of terminology and understanding by incorporating levels of causation. The importance of dividing behavior into levels of influence is probably dependent on the interest of the researcher. Based on an initial study on TTI by its developers Petraitis et al. [21], the authors felt that some readers might disagree with the location of some variables within the levels of causation. To resolve the issue, the authors specified that the location of specific variables only affected the order in which findings were discussed and had no effect on conclusions drawn i.e. the relationship between peer drug use and illicit substance use, is the same whether peer drug use is considered an immediate or a distal influence.

In summary, the literature in this review provided information that compares and contrasts the SEM and TTI. However, the quality of the studies used in this review is compromised due to the lack of some information in the articles assessed. The data that is not reported may be attributed to some of the studies following a secondary study design which does not elaborate much on methodology. In addition, some studies involved the secondary analysis of primary data from another study or database and therefore did not effectively elaborate on how the primary data was obtained. Finally, a more precise assessment of the extent that screening and vaccination differ was not a factor that could clearly be assessed by the available articles due to the lack of relevant studies covering those topics of interest.

### Limitations

A number of potential limitations may have affected the validity of our results. Mostly in terms of possible publication and selection biases as a result of having more strict inclusion criteria for one group of studies compared to the other. Despite the efforts to identify all relevant studies, it is possible that some may have been missed. Furthermore, because studies considered for inclusion were English language only, further data might have been excluded.

In spite of these limitations, the main strength of this study is that it assessed two multi-level models that researchers rarely have a chance to apply in research. This paper has outlined how these models have been implemented.

## Conclusions

This review presented information on two multi-level prevention models, the SEM and TTI regarding screening and vaccination. The findings obtained in this review pointed to some general conclusions about the extent that screening and vaccination as determined by the SEM and TTI are different. In general, the theoretical constructs, concepts and composition of the models are similar and the main difference is in structure and variable interaction. Additionally, the TTI is more specific to its application, compared to the SEM which tends to be more flexible.

This review has identified key considerations for potentially successful prevention programs. Since the SEM and TTI are similar in composition, the following points can be considered when selecting a model:

1. Information: If you think it is essential to divide the information you obtain into levels of causation then the TTI is best. The SEM does not consider levels of causation. Consider depth of the information you want.
2. Target group: The SEM differentiates the levels of society compared to the TTI that combines the higher levels of society into one group. Consider who you want information from.
3. Availability of information: In general, studies that lacked adequate information failed to conceptualize the TTI. Consider how much information is available to you and ease of obtaining the data.
4. Resources: If you have adequate resources and time to conduct a longitudinal study, then the TTI would be a good option for research. Longitudinal studies have been proven to be more effective for research applying the TTI compared to cross-sectional studies. Consider the resources and time you have for the research.

Finally, when it comes to effectiveness, the SEM appears to be effective for screening and vaccination studies. Perhaps this is due to the fact that it has been applied more in prevention method studies compared to the TTI. Notwithstanding that many authors claim that the model they applied (either SEM of TTI) was effective, the empirical basis of such conclusions is inadequately explained in the articles we studied. In many studies, information about data collection, sampling, and data analysis were at best incomplete and often even lacking. These findings highlight the emerging nature of this research area and the need for more research to be conducted.

## Additional files

Additional file 1: Figure S1. Social Ecological Model. Description of data: An illustration of the Social Ecological Model [22]. (DOCX 123 kb)
Additional file 2: Figure S2. Theory of Triadic Influence. Description of data: An illustration of the Theory of Triadic Influence [7]. (DOCX 358 kb)
Additional file 3: Data Extraction Form. Description of data: The Data Extraction Form used for this systematic review. (DOCX 71.2 kb)
Additional file 4: Table S1. Studies awaiting classification which not included in this review because on inaccessibility; **Table S2**: Characteristics of included SEM studies; **Table S3**: Characteristics of included TTI studies; **Table S4**: Quality assessment of studies using the SEM; **Table S5**: Quality assessment of studies using the TTI. Description of the data: Tables with study results [23–104]. (DOCX 132 kb)

## Abbreviations

CAQDA: Computer Assisted/Aided Qualitative Data Analysis Software
CRQ: Central Research Question
DEF: Data Extraction Form
NA: Not applicable
NR: Not reported
PSA: Prostate Specific Antigen
SEM: Social Ecological Model
SES: Social Economic Status
TTI: Theory of Triadic Influence
USA: United States of America
