# Supplementary material for: Screening and vaccination as determined by the Social Ecological Model and the Theory of Triadic Influence: a systematic review
Source: BMC Public Health. 2016 Nov 17;16:1166. doi: 10.1186/s12889-016-3802-6 (PMC5114823; doi:10.1186/s12889-016-3802-6)
Supplement: Additional file 1: Figure S1. — Social Ecological Model. Description of data: An illustration of the Social Ecological Model [22]. (DOCX 123 kb) [file 12889_2016_3802_MOESM1_ESM.docx]

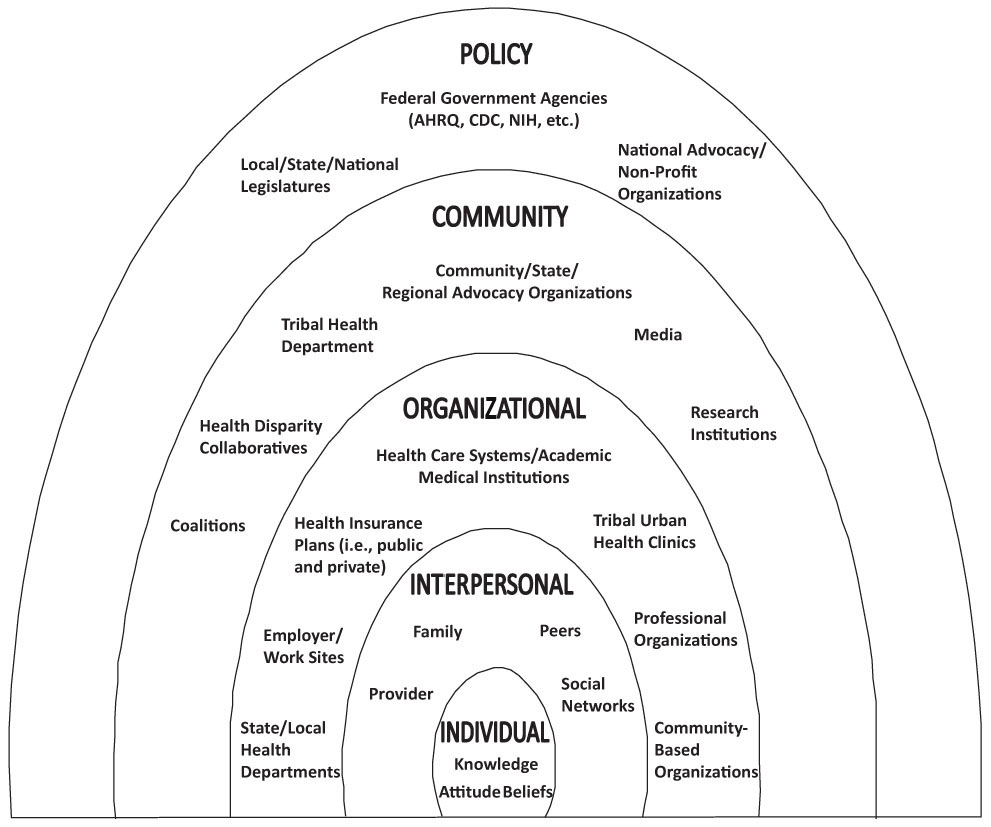


**Figure S1:** Social Ecological Model (CDC, 2013). Published in accordance with the non-copyright laws of the Centers of Disease Control and Prevention
