# Supplementary material for: Screening and vaccination as determined by the Social Ecological Model and the Theory of Triadic Influence: a systematic review
Source: BMC Public Health. 2016 Nov 17;16:1166. doi: 10.1186/s12889-016-3802-6 (PMC5114823; doi:10.1186/s12889-016-3802-6)
Supplement: Additional file 2: Figure S2. — Theory of Triadic Influence. Description of data: An illustration of the Theory of Triadic Influence [7]. (DOCX 358 kb) [file 12889_2016_3802_MOESM2_ESM.docx]

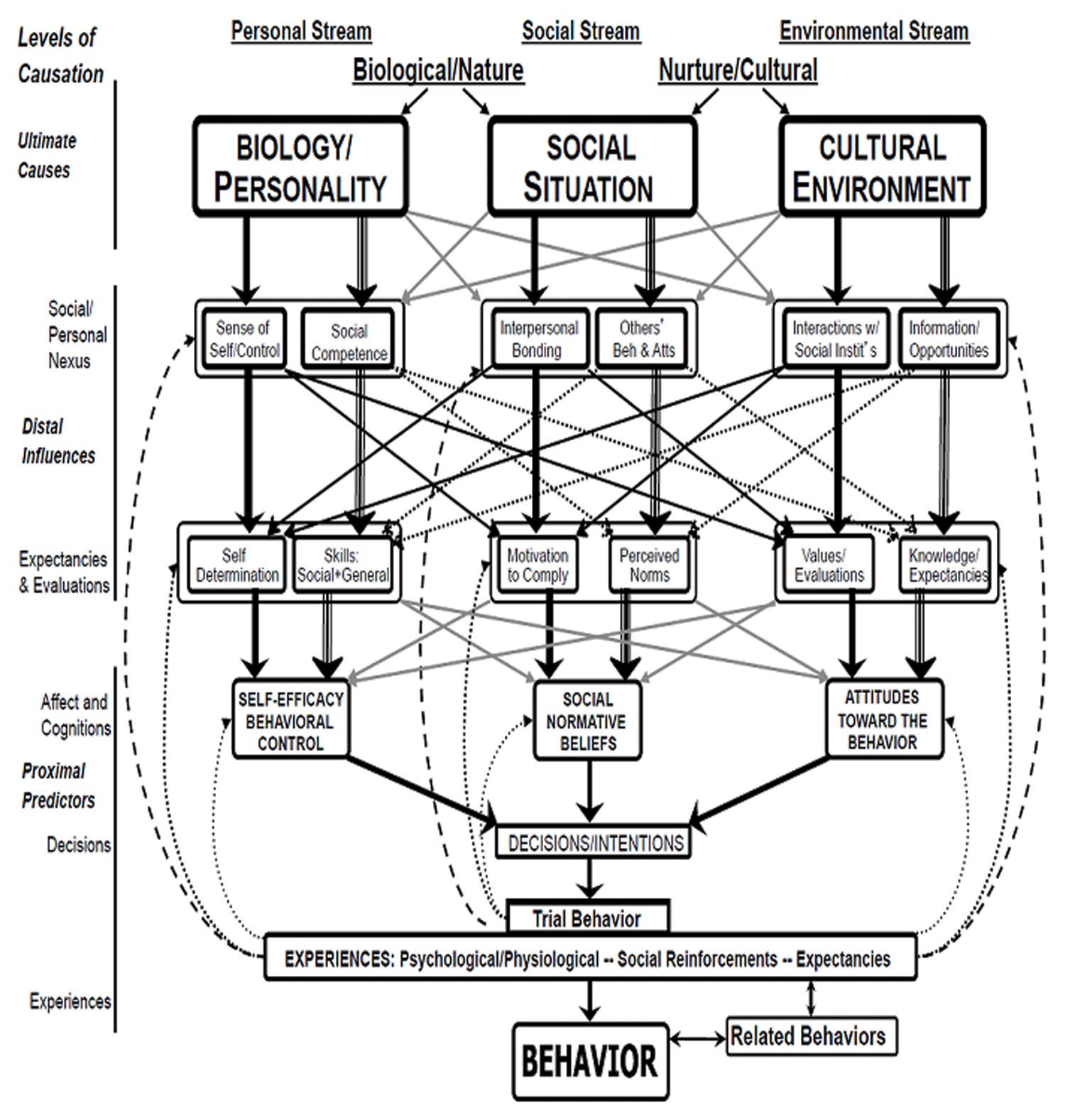


**Figure S2:** Theory of Triadic Influence (Flay et al, 2009). Published with permission from John Wiley and Sons through Copyright Clearance Center.
