# Supplementary material for: Screening and vaccination as determined by the Social Ecological Model and the Theory of Triadic Influence: a systematic review
Source: BMC Public Health. 2016 Nov 17;16:1166. doi: 10.1186/s12889-016-3802-6 (PMC5114823; doi:10.1186/s12889-016-3802-6)
Supplement: Additional file 3: — Data Extraction Form. Description of data: The Data Extraction Form used for this systematic review. (DOCX 71.2 kb) [file 12889_2016_3802_MOESM3_ESM.docx]

Data Extraction Form

## GENERAL INFORMATION

| ID#: | | | Date form completed: | | | Excluded  Included | |
| --- | --- | --- | --- | --- | --- | --- | --- |
| Title: | | | | | | | |
| Author(s): | | | | | | | |
| Publication type | | Full text article / Abstract / Dissertation / Progress report / Letter/ Book  Other | | | | | |
| Source of information | | | | | | | |
| Year: | Volume: | | | Issue: | Page(s): | | Country: |
| Journal title: | | | | | #of citations Web of Science: | | |

## INCLUSION/EXCLUSION CRITERIA

| Geography | Include any country. Priority given to Sub-Saharan African countries. |
| --- | --- |
| Time | Include studies starting from the year 2000 to date. |
| Participants | Includes all people with health behavior affected from intrapersonal, interpersonal, organizational, community and policy level, in accordance with the SEM.  Or the TTI where behavior is influenced from the ultimate, distal, and proximal levels. As well as the intrapersonal, interpersonal social and socio-cultural environment streams. |
| Disease | Priority given to cancers and diseases that can be screened or vaccinated against. Also include substance abuse/risk behavior if they illustrate the use of the TTI. |
| Exposure/Intervention | Primary and secondary prevention procedures including vaccination, screening methods and control of risk behavior. |
| Comparison | May not be applicable in this study but will include people who do not practice primary and secondary prevention measures |
| Study Model | Include Social Ecological Model and Theory of Triadic Influence |
| Outcome | Set of optimal preventive measures by SEM and set of measures by TTI |
| Language | English |

## STUDY ELIGIBILITY

| FACTORS | ASSESSMENT | Location in text or source *(pg & ¶/fig/table)* |
| --- | --- | --- |
| STUDY MODEL |  |  |
| Social Ecological Model | Yes  No  Unclear |  |
| Theory of Triadic Influence | Yes  No  Unclear |  |
| PARTICIPANTS AND SETTING |  |  |
| Are the participants of the study human? | Yes  No  Unclear |  |
| Is the study located in sub-Saharan Africa? | Yes  No  Unclear |  |
| Is the disease under study cancer or any disease or condition that can be screened or vaccinated or a risk behavior? | Yes  No  Unclear |  |
| INTERVENTIONS |  |  |
| Primary prevention (Vaccination, health education) | Yes  No  Unclear |  |
| Secondary Prevention (Screening, medical treatment) | Yes  No  Unclear |  |
| OUTCOMES |  |  |
| Conclusions? | Present  Missing |  |
| FINAL DECISION | Include  Exclude |  |

## REASONS FOR EXCLUSION OF STUDY FROM REVIEW

| What are the reason(s) for exclusion? | Different theory/model used  Non-human participants  irrelevant disease/condition  irrelevant intervention  irrelevant outcomes assessed  Duplicate publication  Other |
| --- | --- |

**DO NOT PROCEED IF STUDY EXCLUDED FROM REVIEW**

# CHARACTERISTICS OF INCLUDED STUDIES

## Part 1: GENERAL STUDY DETAILS

|  | **Descriptions as stated in report/paper** | **Location in text or source** *(pg & ¶/fig/table)* |
| --- | --- | --- |
| Study aims | To explore views (attitudes, practices, knowledge and beliefs) of vaccination, screening and disease/condition  To explore the promotion of vaccination, screening or treatment  To examine participation/acceptance and non- acceptance of vaccination, screening or treatment  To determine the cause of behavior  To form/evaluate interventions  Other |  |
| Research Question(s) | Yes  No  Unclear  Not reported |  |
| Conclusion addressing RQ | Yes  No  Unclear  Not reported |  |
| Hypothesis | Yes  No  Unclear  Not reported |  |
| Conclusion addressing Hypothesis | Yes  No  Unclear  Not reported |  |
| Study setting | High income country/ Middle income country/ Low income country/ National/ Urban/ Rural/ Region/ Province (state)/ County/ City (town)/ Community/  Unclear Other Not reported |  |
| Unit of Observation | Individual/Intrapersonal (patient)/  Interpersonal/ social situation (family, friends, peers)/ Organizational (Churches, stores, community orgs.)/ Community (Social networks)/ Policy/National (Local, state, federal)  Unclear Other |  |
| Level of Analysis | Individual/Intrapersonal (patient)/  Interpersonal/ social situation (family, friends, peers)/ Organizational (Churches, stores, community orgs.)/ Community (Social networks)/ Policy/National (Local, state, federal)  Unclear Other |  |
| Target Disease | Bladder Cancer/ Breast Cancer / Colon and Rectal Cancer/ Endometrial Cancer/ Kidney Cancer/ Leukemia/ Lung Cancer/ Liver Cancer/ Melanoma/ Non-Hodgkin Lymphoma/ Pancreatic Cancer/ Prostate Cancer/ Thyroid Cancer/ Cancer (general)  Cervical Cancer  Drug/substance abuse  Other |  |
| Category of treatment investigated | Screening  Vaccination  Substance abuse/risk behavior intervention  Other |  |

## Part 2: EVALUATION DESIGN

|  | **Descriptions as stated in report/paper** | **Location in text or source** *(pg & ¶/fig/table)* |
| --- | --- | --- |
| Type of Study | Qualitative  Quantitative  Mixed  Unclear |  |
| Primary  research | Randomized Controlled Trial/ Nonrandomized intervention/ Case–control/ Case study/ Cross-sectional/Descriptive/ Correlation/ Longitudinal  / Cohort  Unclear Other  N/A |  |
| Secondary  research | Metaanalysis /Systematic review /Simple overview /Guideline  Unclear Other  N/A |  |
| INCLUSION CRITERIA | |  |
| Independent variables | Constructs of the TTI by Petraitis & Flay (1994/95, 2009)  Constructs of the TTI other  Constructs of the TTI not referenced  Constructs of the SEM by Sweat and Denison (1995)  Constructs of the SEM by McLeroy (1988)  Constructs of the SEM by Stokols(1992, 2003)  Constructs of the SEM by Bronfenbrenner (1979/89, 2004)  Constructs of the SEM other  Constructs of the SEM not referenced |  |
| Independent variables TTI | intrapersonal stream (demographics, biology and personality that influence self-efficacy)  social situation/context stream (interpersonal/normative, characteristics in an individual’s immediate social setting(s) that influence behavioral norms)  socio-cultural environment stream (attitudinal, broader social and cultural environmental factors that influence attitudes toward a behavior)  ultimate causes (demographics, individual has the least control over)  distal influences (fear, self-efficacy)  proximal predictors (support, response, severity)  immediate precursors (individual has most control over)  Other  Unclear  Not reported  Not Applicable |  |
| Independent variables SEM | Intrapersonal/Individual level (demographics, knowledge, attitude, perceptions, self-efficacy)  Micro-system/Interpersonal level (peers, family, school, work)  Mesosystem (interrelation between microsystems)  Exosystem (enviro. settings causing indirect influence)  Macrosystem (culture, political systems, societies)  Organizational/Institutional level (Social institutions and organization characteristics, formal/informal rules for operations)  Community level (relationships among organization, institutions and informal networks with defined boundaries)  Public Policy (Local, State and national laws and policies)  Technological level (treatment/vaccines etc.)  Relational/Dyadic (family, couple, may also include household, community)  Environmental (physical/social changes in environment)  Structural (laws and policies)  Super-structural (social justice, class, race, gender, equity)  Other  Unclear  Not reported  Not Applicable |  |
| Is it a modified/incomplete version of the SEM or TTI? | Yes  No  Unclear |  |
| If it is modified, how? | Different number of levels/constructs from that of the original author  Different names/contents  Incorporates another model/theory  Other  Not Applicable |  |
| Demographic Data  (requirements for participation) | Sex/ Age/ Race/ Social Economic Status/ Health Status/Practices/ Education Level/ Religion/  Marital Status  Other Not Applicable |  |

## Part 3: DATA SOURCES 1 – FACILITATORS

Repeat section if more than one type of facilitator was used. If the study did not use facilitators skip to Part 4.

|  | **Descriptions as stated in report/paper** | **Location in text or source** *(pg & ¶/fig/table)* |
| --- | --- | --- |
| Source/ Target Population | Government/ Parents, Guardians / Healthcare Professionals/facilities/ Community leaders/sites (churches, workplace)/ Researchers, Mediators/ Educational institutions (teachers, schools) / Databases (online, offline)  Other Not applicable |  |
| Sample size | No.: |  |
| Withdrawals and exclusions | Yes  No  Unclear  Not reported  Not applicable |  |
| Follow-up | No / Yes /Unclear/ Not applicable Not reported |  |
| SAMPLING | |  |
| Sampling Design/Strategy | Convenience /Judgmental /Quota /Snowball /  Simple Random /Systematic /Stratified /Cluster  Other  Not reported Not applicable |  |
| Method of recruitment of participants | Phone/ Mail / Advertisement (Posters, Brochures)/Word of Mouth/ Other  Not reported Not applicable |  |

## Part 4: DATA SOURCES 2 – ACTUAL PARTICIPANTS

Repeat section if more than one type of participant participated.

|  | **Descriptions as stated in report/paper** | **Location in text or source** *(pg & ¶/fig/table)* |
| --- | --- | --- |
| Source/ Target Population | Men/ Women/ Adults/ Youth/ Adolescents/ Children/ Policymakers/ Parents and Guardians/  Doctors/  Patients/ Nurses / Other Healthcare Professionals / Community leaders / Teachers/ Students/ Book, journal articles  Other Not applicable |  |
| Sample size (final study participants) | No.: |  |
| Withdrawals and exclusions | Yes  No  Unclear  Not reported  Not applicable |  |
| Follow-up | No / Yes /Unclear/Not reported/Not applicable |  |
| Min. age | No.: |  |
| Max. age | No.: |  |
| Mean age | No.: |  |
| Sex | Male  Female  Not reported  Not applicable |  |
| Race | Black  White/Caucasian  Hispanic/Latino  Asian  Arabic  Native  Mixed/biracial  Other  Not reported  Not applicable |  |
| Social Economic Status | Upper-class  Middle-class  Lower-class  Other  Not reported  Not applicable |  |
| Education Level | Higher than secondary/ Degree education  Secondary/ high school  Primary school and less  Not reported  Not applicable |  |
| Religion | Christian Protestant/Catholic  Muslim  Hindu  Jewish  None  Other  Not reported  Not applicable |  |
| Marital Status | Married/Cohabiting/Dating  Single/Never Married  Divorced/Separated  Not reported  Not applicable |  |
| Vaccination Status | Not vaccinated  Vaccinated  Incomplete vaccination  Mixed vaccinated and not vaccinated  Not applicable  Not reported |  |
| Screening Status | Not screened  Screened  Mixed screened and not screened  Not applicable  Not reported |  |
| SAMPLING | |  |
| Sampling Design/Strategy | Convenience /Judgmental /Quota /Snowball /  Simple Random /Systematic /Stratified /Cluster  Other Not reported Not applicable |  |
| Method of recruitment of participants | Phone/ Mail / Advertisement (Posters, Brochures)/Word of Mouth/  Other Not reported Not applicable |  |
| Informed consent obtained | Yes  No  Unclear  Not reported  Not applicable |  |
| COLLECTION | |  |
| Data Collection Method/Tools | Observation /Questionnaires /Standardized tests /Interviews /Focus groups /Diaries /Think-a-loud protocols /Physical and biophysical measurements /System measurements /Secondary data  Other/ Not reported/Not applicable |  |
| Data collection tool | Developed by the researchers  Based on another study  Unclear  Not reported |  |
| Reliability check performed e.g. Cronbach alpha (α) | Yes  No  Unclear  not reported  Not applicable |  |
| MAIN OUTCOME MEASURE(S) | |  |
| Dependent Variables | Screening practiced  Vaccination practiced  Screening not practiced  Vaccination not practiced  Non-risk behavior, Rehabilitation, therapy practiced  Smoking, Drug, risk behavior practiced  Other  Not reported |  |

## Part 5: ANALYSIS AND EVALUATION

|  | **Descriptions as stated in report/paper** | **Location in text or source** *(pg & ¶/fig/table)* |
| --- | --- | --- |
| Analysis Plan - Qualitative | Grounded theory /Constant comparison /Phenomenological approach /Thematic content analysis /Framework analysis /Content analysis  Other  Not reported  Not applicable |  |
| Effect of intervention | Totally effective  Partly effective  Ineffective  Unclear  Not reported  Not applicable |  |
| Analysis Plan -Statistical Methods | Chi-square /Mann-Whitney U-Test /Regression /ANOVA /Baron and Kenny’s test /Sobel test /Descriptive statistics /T-tests/ Wilcoxon test /Correlation/ Z test/ Tailed tests  Other Not reported Not Applicable |  |
| **Bias** | Selection bias/ Information/measurement/observer bias/ Interviewer/ Recall/Report Migration / Confounding  Other  Unclear  Not Reported |  |

## Part 6: RESULTS AND EVALUATION

|  | **Descriptions as stated in report/paper** | **Location in text or source** *(pg & ¶/fig/table)* |
| --- | --- | --- |
| OUTCOMES ASSESSED | |  |
| Positive Predictors of practicing Screening/Vaccination  /Intervention and not using drugs or following risk behavior | Positive influences and surroundings i.e. family, peers, community involvement  Personal beliefs  Recommendation from health care provider(s)  Access to health care providers, facilities  Health knowledge, information, awareness  Having health insurance  Polices/Law enforcing behavior  Medical status/practices  Culture  Other  Not Reported |  |
| Negative Predictors of i.e. causing people not to practice Screening/Vaccination  /Intervention, and using drugs/practicing risk behavior | Negative influences and surroundings i.e. family, peers, community involvement  Negative personal beliefs (fear, nervous, painful, wrongly informed)  Lack of recommendation from health care provider(s)  Lack of access to health care providers, facilities  Lack of knowledge, information, awareness  Lack of health insurance  Polices/Law  Medical status/practices  Culture  Other  Not Reported |  |
| Overall view of model | SEM/TTI is good model  SEM/TTI is not a good model  SEM/TTI is better if modified  Not Reported |  |
| STATISTICAL RESULTS | |  |
| Effect Size | Odds Ratio  Relative Risk  Relative risk reduction  Absolute risk reduction  Not Reported  Not applicable |  |
| Power calculation | Significance criterion (p-values) specified  Alpha error (α) specified  Beta error (β) specified  Power calculation  Not Reported  Not applicable |  |

## Part 7: OTHER INFORMATION

|  | **Descriptions as stated in report/paper** | **Location in text or source** *(pg & ¶/fig/table)* |
| --- | --- | --- |
| Ethical approval needed/ obtained for study | Yes  No  Unclear  Not Reported |  |
| Funding Sources | Industry/Organization/ Public/ mixed/ travel / self-funded / educational institute/ other  Unclear  Not Reported |  |
| References to other relevant studies | Present  Missing |  |
| Correspondence required for further study information | Yes  No  Unclear |  |
