# Supplementary material for: Screening and vaccination as determined by the Social Ecological Model and the Theory of Triadic Influence: a systematic review
Source: BMC Public Health. 2016 Nov 17;16:1166. doi: 10.1186/s12889-016-3802-6 (PMC5114823; doi:10.1186/s12889-016-3802-6)
Supplement: Additional file 4: Table S1. — Studies awaiting classification which not included in this review because on inaccessibility; Table S2: Characteristics of included SEM studies; Table S3: Characteristics of included TTI studies; Table S4: Quality assessment of studies using the SEM; Table S5: Quality assessment of studies using the TTI. Description of the data: Tables with study results [23–104]. (DOCX 132 kb) [file 12889_2016_3802_MOESM4_ESM.docx]

**Table S1: Studies awaiting classification which not included in this review because on inaccessibility**

| **Study**  **(author(s), year)** | **Study Model** | **Participants** | **Setting** | **Disease or Condition** | **Intervention** | **Outcomes** |
| --- | --- | --- | --- | --- | --- | --- |
| **Possible SEM studies** | | | | | | |
| Eastern Nursing Research Society 27th Annual Scientific Sessions Abstracts.  (ENRS, 2015) | Unclear | Human | Unclear | Unclear | Unclear | Present |
| The Heart of the Matter: Reducing CVD Risk.  (Hayman LL, et al.; 2009) | Unclear | Unclear | Unclear | Cardiovascular Disease | Unclear | Missing |
| Screening for Psychosocial Risk at Pediatric Cancer Diagnosis: The Psychosocial Assessment Tool.  (Kazak AE, et al.; 2011) | Unclear | Human | Unclear | Cancer | Screening | Present |
| Community Participation in Health Initiatives for Marginalized Populations.  (Larson C, et al.; 2009) | SEM | Human | Unclear | Unclear | Unclear | Present |
| Factors Influencing the Prostate Cancer Screening Behavior in African American Men.: Dl.  (Song L, et al.; 2009) | Unclear | Human | USA | Cancer | Screening | Missing |
| **Possible TTI studies** | | | | | | |
| Why do Some Teens Turn to Drugs? A Focus Group Study of Drug Users' Experiences.  (Nakhaee N, et al.; 2009) | Unclear | Human | Iran | Risk behavior | None | Present |

**Table S2: Characteristics of included SEM studies**

| **Study**  **(author(s), year)** | **Aim** | **Study Location** | **Target disease/ condition, treatment** | **Study Type** | **Model, Variables** | **Participants** | **Analysis** | **Outcomes** |
| --- | --- | --- | --- | --- | --- | --- | --- | --- |
| Factors Contributing to Filipinos' Resistance to Preventive Screening.  (Atassi K, et al.; 2010) | Examine acceptance and non- acceptance of vaccination, screening or treatment | National  Philippines | D: Colon and Rectal Cancer  T: screening | Mixed methods  Unclear primary study  Systematic review | SEM not referenced | Facilitator(s)  1. Databases - Sample size 5  2. Government - Sample size 3  Participant(s)  1. Books, journals  Sample size 5;  Data collection method Secondary data;  2. Other  Data collection method Interviews; | NA | Positive predictors - positive influences and surroundings  Negative predictors - negative influences and surroundings  Overall view of model - good |
| Factors Predicting BCG Immunization Status in Northern Nigeria: A Behavioral-ecological Perspective.  (Babalola S & Lawan U; 2009) | Determine the cause of behavior | Nigeria | D: Other  T: Vaccination | Unclear primary study  Other secondary study | SEM by McLeroy  Not modified | Inclusion criteria - other  Facilitator(s)  1. Databases - sample size 1;  Participant(s)  1. Women  Sampling strategy simple random, stratified;  Collection method interview and secondary data; reliability check NR;  Outcome measure vaccination practiced and vaccination not practiced  2. Men  Sampling strategy snowball;  Data collection method interview and secondary data; reliability check NR;  Outcome measure: vaccination practiced and vaccination not practiced  3. Healthcare professionals  Sample size 255;  Data collection method secondary data; reliability check NR;  Outcome measure: other | Regression | Positive predictors - positive influences and surroundings, healthcare provider recommendations, access to healthcare providers/facilities, knowledge/awareness, policies/rules, medical status/practices, other  Negative predictors - lack of access to healthcare providers/facilities, other  Overall view of model - good |
| Enhancing Benefits or Increasing Harms: Community Responses for HIV among Men Who have Sex with Men, Transgender Women, Female Sex Workers, and People Who Inject Drugs.  (Baral S, et al.; 2014) | Other | Middle income  Low income  Multiple countries | D: Other  T: Other | Mixed methods  Systematic review | SEM by other author  Modified version - different number of levels | Inclusion criteria - other  Facilitator(s)  1. databases  Sample size 1;  Participant(s)  1. Books, journal articles  Sample size 22;  sampling strategy judgmental;  Data collection method other; reliability check NR;  Outcome measure: other | NR | Positive predictors - positive influences and surroundings, access to healthcare providers/facilities, policies/rules  Negative predictors - negative influences and surroundings, negative personal beliefs, lack of access to healthcare providers/facilities, lack of knowledge/awareness, policies/rules  Overall view of model - NR |
| Understanding the Interplay of Factors Informing Vaccination Behavior in Three Canadian Provinces.  (Boerner F, et al; 2013) | Examine acceptance and non- acceptance of vaccination, screening or treatment | Canada | D: Other  T: vaccination | Mixed methods  Case study | SEM by other author  Modified version - different names/contents ; incorporates another model | Inclusion criteria - age, SES, education, marital, other  Facilitator(s)  1. other  Participant(s)  1. Other  Sample size 143;  min age 18; male and female;  upper, middle, lower SES; degree, secondary, primary education; mixed vaccinated and not vaccinated;  Sampling strategy judgmental and stratified;  Data collection method questionnaire and focus groups; reliability check NR;  Outcome measure: vaccination practiced and vaccination not practiced | Thematic analysis  Framework analysis  Descriptive | Positive predictors - positive influences and surroundings, personal beliefs, other  Negative predictors - negative influences and surroundings, negative personal beliefs, lack of knowledge/awareness, other  Overall view of model - good |
| Theorizing Social Context: Rethinking Behavioral Theory  (Burke NJ, et al.; 2009) | Other | USA | D: Breast cancer  T: Screening | Qualitative  Unclear primary study  Simple overview | SEM by Stokols  Not modified | Participant(s)  1. Women  Ethnicity Latin, Asian;  Outcome measure: screening practiced | NA | Positive predictors - NR  Negative predictors - NR  Overall view of model - good |
| Willingness to Participate in HIV Vaccine Trials among Men Who have Sex with Men in Chennai and Mumbai, India: A Social Ecological Approach  (Chakrapani V, et al.; 2012) | Examine acceptance and non- acceptance of vaccination, screening or treatment | India | D: Other  T: vaccination | Qualitative  Unclear primary study | SEM not referenced | Inclusion criteria - sex, age, other  Facilitator(s)  1. community leaders/sites  Participant(s)  1. Men  Sample size 68;  min age 20, max age 46, mean age 28;  vaccinated;  Sampling strategy judgmental and stratified;  Data collection method focus groups; reliability check unclear;  Outcome measure: vaccination practiced  2. Other  Sample size 14;  min age 29, max age 60; mean age 40;  Sampling strategy judgmental;  Data collection method interview; reliability check unclear;  Outcome measure: vaccination practiced | Constant comparison  Thematic analysis | Positive predictors - positive influences and surroundings, healthcare provider recommendations, access to healthcare providers/facilities, knowledge/awareness, insurance, other  Negative predictors - negative influences and surroundings, negative personal beliefs, lack of healthcare provider recommendations, lack of access to healthcare providers/facilities, lack of knowledge/awareness, medical status/practices, other  Overall view of model - good |
| American Heart Association Childhood Obesity Research Summit Report.  (Daniels RS, et al.; 2009) | Other | USA | D: Other  T: Screening  other | Simple overview | SEM not referenced | Inclusion criteria - age | NA | Positive predictors - NR  Negative predictors - NR  Overall view of model - good |
| Perspectives of African American, Amish, Appalachian and Latina women on Breast and Cervical Cancer Screening: Implications for Cultural Competence.  (Documét PI, et al.; 2008) | Determine the cause of behavior  Other | Urban  Rural  USA | D: Cancer (breast, cervical)  T: screening | Qualitative  Case study | SEM by other author  Modified version - incorporates another model | Inclusion criteria - sex, race  Facilitator(s)  1. Researchers, mediators - sample size 12  2. databases - sample size 4  Participant(s)  1. Women  Sample size 210 and then 102; follow-up was conducted;  Ethnicity black, white, Latin, other race;  Data collection method focus groups; reliability check performed;  Outcome measure: screening practiced  2. Healthcare professionals  Sample size 168; follow up was conducted;  degree education;  Sampling strategy judgment, simple random and stratified;  Data collection method questionnaires and interviews; reliability check performed;  Outcome measure: screening practiced | Thematic analysis  Framework analysis | Positive predictors - positive influences and surroundings, healthcare provider recommendations, knowledge/awareness, policies/rules, culture  Negative predictors - negative personal beliefs, lack of healthcare provider recommendations, other  Overall view of model - NR |
| Increasing Use of Mammography among Older, Rural African American Women: Results from a Community Trial.  (Earp JA et al, 2002) | Form or evaluate interventions | Rural  USA | D: Breast cancer  T: Screening | Non- randomized intervention  Cohort | SEM by McLeroy  Not modified | Inclusion criteria - sex, age, race, SES, health  Participant(s)  1. Women  Sample size 801; follow up was conducted;  min age 50;  Ethnicity - black race; mixed screened and not screened;  Sampling strategy simple random, systematic;  Data collection method interview; reliability check performed;  Outcome measure: screening practiced | Framework analysis  Chi-square  Regression  Correlation  Other | Positive predictors - healthcare provider recommendations, other  Negative predictors - NR  Overall view of model - NR |
| Beyond Effectiveness: Evaluating the Public Health Impact of the WISEWOMAN Program.  (Farris RP, et al.; 2007) | Form or evaluate interventions | USA | D: Other  T: Screening  other | Unclear primary study  Other secondary study | SEM not referenced  Modified version - incorporates another model | Inclusion criteria - other  Participant(s)  1. Other  Sample size 14;  Data collection method secondary data;  Outcome measure: other | Correlation | Positive predictors - NR  Negative predictors - NR  Overall view of model - NR |
| Barriers and Facilitators to HPV Vaccination of Young Women in High-income Countries: A Qualitative Systematic Review and Evidence Synthesis.  (Ferrer HB, et al.; 2014) | Examine acceptance and non- acceptance of vaccination, screening or treatment | Multiple countries | D: Cervical cancer  T: vaccination | Qualitative  Systematic review | SEM by McLeroy  Not modified | Inclusion criteria - sex, age, health, other  Facilitator(s)  1. Databases - Sample size 6  Participant(s)  1. Books/journal articles  Sample size 41;  Sampling strategy judgmental;  Data collection method secondary data and other;  Outcome measure: vaccination practiced and vaccination not practiced | Thematic analysis | Positive predictors - positive influences and surroundings, personal beliefs, healthcare provider recommendations, policies/rules  Negative predictors - negative personal beliefs, lack of healthcare provider recommendations, lack of insurance, culture, other  Overall view of model - NR |
| Behavioral Science Research in the Prevention of Diabetes : Status and Opportunities.  (Fisher EB, et al.; 2002) | Explore the promotion of vaccination, screening or treatment  Other | NR | D: Other  T: Screening  Substance abuse/risk behavior | Simple overview | SEM not referenced | Participant(s)  1. Books, journals  Data collection method secondary data; | NA | Positive predictors - positive influences and surroundings, knowledge/awareness  Negative predictors - negative influences and surroundings, negative personal beliefs  Overall view of model - NR |
| Applying core principles to the design and evaluation of the 'Take Charge. Take the Test' campaign: what worked and lessons learned.  (Fraze JL et al.., 2009) | Explore views on vaccination, screening or disease/condition  Examine acceptance and non- acceptance of vaccination, screening or treatment  Form or evaluate interventions | USA | D: Other  T: screening | Case study | SEM by other author  Modified version - different names/contents ; incorporates another model | Inclusion criteria - sex, age, race, SES, health, education, marital, other  Facilitator(s)  1. Healthcare professionals/facilities  2. Community leaders/sites - sampling strategy judgmental;  Participant(s)  1. women  Ethnicity black; lower SES;  Data collection method observation, interview; reliability check NR;  Outcome measure: other  2. other  Data collection tool interview; reliability check NR;  Outcome measure: other | Qualitative NR  Quantitative Other | Positive predictors - knowledge/awareness  Negative predictors - other  Overall view of model - NR |
| "I Connect with the Ringleader:" Health Professionals' Perspectives on Promoting the Sexual Health of Adolescent Males.  (Garcia MC, et al.; 2014) | Explore the promotion of vaccination, screening or treatment | Urban  USA | D: Other  T: Intervention Other | Qualitative  Unclear primary study | SEM not referenced | Inclusion criteria - other  Facilitator(s)  1. Healthcare professionals/facilities - Sample size 1;  Participant(s)  1. Other  Sample size 9; male and female;  Sample strategy judgmental;  Data collection method interviews; reliability check NR;  Outcome measure: other | Qualitative other | Positive predictors - positive influences and surroundings, access to healthcare providers/facilities, knowledge/awareness, medical status/practices, other  Negative predictors - negative influences and surroundings, negative personal beliefs, lack of knowledge/awareness  Overall view of model - NR |
| Health Disparities in Colorectal Cancer Screening in the United States: An Application of the Social Ecological Model  (Greene DM; 2011) | Form or evaluate interventions | National  USA | D: Colon and Rectal Cancer  T: Screening | Simple overview | SEM by McLeroy  Modified version - different names/contents | Participant(s)  1. Books, journal articles  Data collection method secondary data; | NA | Positive predictors - positive influences and surroundings, knowledge/awareness, insurance  Negative predictors - negative influences and surroundings, negative personal beliefs, lack of access to healthcare providers/facilities, other  Overall view of model - good |
| Social Context and Drivers of Intimate Partner Violence in Rural Kenya: Implications for the Health of Pregnant Women.  (Hatcher AM, et al.; 2013) | Determine the cause of behavior  Form or evaluate interventions | Rural  Kenya | D: Other  T: Screening | Qualitative  Unclear primary study | SEM by other author  Modified version - different number of levels | Inclusion criteria - health  Facilitator(s)  1. Healthcare professionals/facility - sample size 4; sampling strategy judgmental;  2. Community leaders/sites - sample size 2; sampling strategy judgmental;  Participant(s)  1. Women  Sample size 39;  min age 18, max age 35;  Sampling strategy convenience;  Data collection method focus groups; reliability check NR;  Outcome measure: other  2. Men  Sample size 32; min age 18, max age 59;  Data collection method focus groups; reliability check NR;  Outcome measure: other  3. Other  Sample size 20;  min age 23, max age 64; male, female;  Sampling strategy judgmental;  Data collection method interviews; reliability check NR;  Outcome measure: other | Grounded theory | Positive predictors - positive influences and surroundings  Negative predictors - negative influences and surroundings, negative personal beliefs, medical status/practices, culture, other  Overall view of model - good |
| Using Technology to Expedite Screening and Intervention for Domestic Abuse and Neglect.  (Hawkins JW, et al.; 2009) | Form or evaluate interventions  Other | USA | D: Other  T: screening | Simple overview | SEM by other author  Not modified | Facilitator(s)  1. Healthcare professionals/facilities  Participant(s)  1. Parents/guardians  Follow up was done;  sampling strategy convenience;  data collection tool questionnaire, interview; reliability check NR;  outcome measure risk no treatment | NA | Positive predictors - NR  Negative predictors - NR  Overall view of model - NR |
| Lay Health Advisors: Promoting Cancer Screening and Reducing Disparities.  ( Hilaire DM; 2011) | Explore the promotion of vaccination, screening or treatment | Rural  USA | D: Cancer  T: screening | Simple overview | SEM by McLeroy  Not modified | Inclusion criteria - sex, race  Participant(s)  1. Books, journal article  Female;  Ethnicity black, Latin, Asian;  Data collection method secondary data;  Outcome measure: screening practiced | NA | Positive predictors - knowledge/awareness, other  Negative predictors - negative personal beliefs, lack of knowledge/awareness  Overall view of model - NR |
| Considerations for National Public Health Leadership in Advancing Sexual Health.  (Ivankovich MB, et al.; 2013) | Other | National  World-wide | D: Other  T: Screening  Vaccination  Substance abuse/risk behavior  other | guideline | SEM not referenced | Participant(s)  1. Book, journal articles  Data collection method secondary data; | NA | Positive predictors - access to healthcare providers/facilities, knowledge/awareness, policies/rules, other  Negative predictors - NR  Overall view of model - NR |
| Chiropractic Care and Public Health: Answering Difficult Questions About Safety, Care Through the Lifespan, and Community Action.  (Johnson, C, et al.., 2012) | Other | Unclear | Disease: Other  Screening  Vaccination  Substance abuse/risk behavior  other | Simple overview | SEM by McLeroy  Not modified | Participant(s)  1. Books, journal articles  Data collection tool secondary data; | NA | Positive predictors - NR  Negative predictors - NR  Overall view of model - good |
| The Social Ecological Model as a Framework for Determinants of 2009 H1N1 Influenza Vaccine Uptake in the United States  (Kumar S, et al.; 2012) | Examine acceptance and non- acceptance of vaccination, screening or treatment | National  USA | D: Other  T: vaccination | Quantitative  Cross-sectional | SEM by McLeroy  Not modified | Inclusion criteria - age, other  Facilitator(s)  1. Other - sample size 1; recruitment other  Participant(s)  1. Adults  Sample size 3689,  min age 18, mean age 46.9; male and female;  Ethnicity black, white, Latin and other; upper, middle, lower SES; degree, secondary and primary education; mixed vaccinated and not vaccinated;  sampling strategy simple random;  Data collection tool questionnaires; reliability check performed;  Outcome measure: vaccination practiced | Regression  t-test  correlation  other | Positive predictors - personal beliefs, healthcare provider recommendations, access to healthcare providers/facilities, insurance, other  Negative predictors - lack of healthcare provider recommendations, lack of knowledge/awareness  Overall view of model - good |
| Issue Editor's Preface: Linkages to Leverage Improvement in Health.  (Larson CO, 2004) | Other | USA | D: Other  T: Screening  Vaccination  Substance abuse/risk behavior  other | Simple overview | SEM by Bronfenbrenner  Not modified | NA | NA | Positive predictors - positive influences and surroundings, healthcare provider recommendations, access to healthcare providers/facilities, knowledge/awareness, other  Negative predictors - NR  Overall view of model - good |
| Public Education and Targeted Outreach to Underserved Women Through the National Breast and Cervical Cancer Early Detection Program.  (Levano W, et al.; 2014) | Other | USA | D: Cancer (breast and cervical)  T: screening | Simple overview | SEM by other author  Not modified | Participant(s)  1. Other  Sample size 5;  Outcome measure: screening practiced; | NA | Positive predictors - access to healthcare providers/facilities, knowledge/awareness, other  Negative predictors - other  Overall view of model - good |
| Theoretical Approaches to Motivating Change: A Farm Family Case Example  (Lund CH, et al.; 2005) | Other | National  Rural  USA | D: Other  T: Other | Simple overview | SEM by other author  Not modified | Participant(s)  1. Men  Sample size 1; age 50; married/dating; screened; outcome measure other;  2. Women  Sample size 1; age 48;  married/dating; screened;  Outcome measure: other  3. Children  Sample size 1;  age 18; male;  secondary education; screened;  Outcome measure: other | NA | Positive predictors - NR  Negative predictors - NR  Overall view of model - good |
| Strategies for Increasing Cervical Cancer Screening Amongst First Nations Communities in Northwest Ontario, Canada.  (Maar M, et al.; 2014) | Examine acceptance and non- acceptance of vaccination, screening or treatment | Canada | D: Cervical cancer  T: screening | Mixed methods  Unclear primary study | SEM by Stokols  Modified version - different number of levels | Inclusion criteria - other  Facilitator(s)  1. Community site/leaders  Participant(s)  1. Other  Sampling strategy judgmental;  Data collection method interview; reliability NR;  Outcome measure: screening practiced | Thematic analysis  Quantitative NR | Positive predictors - positive influences and surroundings, healthcare provider recommendations, knowledge/awareness, medical status/practices, culture, other  Negative predictors - NR  Overall view of model - good |
| Prevalence and Correlates of Breast and Cervical Cancer Screening among a Midwest Community Sample of Low-Acculturated Latinas  (Martínez -Donate AP, et al.; 2013) | Examine acceptance and non- acceptance of vaccination, screening or treatment | USA | D: Cancer (breast and cervical)  T: screening | Cross-sectional | SEM by McLeroy  Modified version - different number of levels | Inclusion criteria - sex, age, race, health status, other  Facilitator(s)  1. Healthcare professionals/facilities  Participant(s)  1. Women  Sample size 278;  min age 18;  Ethnicity Latin/Hispanic; mixed screened and unscreened;  Sampling strategy convenience;  Data collection tool questionnaires; reliability check performed;  Outcome measure: screening practiced and screening not practiced | Chi-square  Regression  Descriptive  Correlation | Positive predictors - healthcare provider recommendations, access to healthcare providers/facilities, knowledge/awareness, other  Negative predictors - negative personal beliefs, lack of access to healthcare providers/facilities, lack of insurance, other  Overall view of model - NR |
| Factors Influencing Frontline Health Service Providersʼ Likelihood to Recommend a Future, Preventive HIV Vaccine to Key Populations in Karnataka, South India  (McClarty LM, et al.; 2015) | Explore views on vaccination, screening or disease/condition  Determine the cause of behavior | India | D: Other  T: vaccination | Mixed methods  Unclear primary study | SEM by McLeroy  Modified version- incorporates another model | Inclusion criteria - age, other  Facilitator(s)  1. Healthcare professionals/facilities - Sample size 7; sampling strategy stratified;  Participant(s)  1. Health care professionals  Sample size 375;  min age 18; male and female;  mixed vaccinated and not vaccinated;  Sampling strategy quota;  Data collection method questionnaire and interviews; reliability check NR;  Outcome measure: vaccination practiced and not practiced | Qualitative NR  Chi-square  Descriptive | Positive predictors - NR  Negative predictors - negative influences and surroundings, negative personal beliefs, lack of healthcare provider recommendations, lack of access to healthcare providers/facilities, lack of knowledge/awareness, other  Overall view of model - NR |
| Barriers and Facilitators to Testing, Treatment Entry, and Engagement in Care by HIV-positive Women of Color.  (Messer LC, et al.; 2013) | Examine acceptance and non- acceptance of vaccination, screening or treatment | USA | D: Other  T: Screening  other | Mixed methods  Unclear primary study | SEM by McLeroy  Modified version - different names/contents | Inclusion criteria - sex, age, race, health  Facilitator(s)  1. healthcare professionals/facilities - Sample size 1;  Participant(s)  1. Women  Sample size 30;  Ethnicity - black, Latin and mixed; not screened;  Sampling strategy convenience and judgmental;  Data collection method interviews, focus groups; reliability check unclear;  Outcome measure: screening practiced and other | Grounded theory  Quantitative NR | Positive predictors - positive influences and surroundings, personal beliefs, healthcare provider recommendations, access to healthcare providers/facilities, knowledge/awareness, medical status/practices, other  Negative predictors - negative influences and surroundings, negative personal beliefs, lack of healthcare provider recommendations, lack of access to healthcare providers/facilities, other  Overall view of model - NR |
| Social Determinants Associated with Colorectal Cancer Screening in an Urban Community Sample of African-American Men  (Mitchell JA, et al.; 2013) | Determine the cause of behavior | High income  USA | D: Colon and Rectal Cancer  T: Screening | Cross-sectional study | SEM by McLeroy  Modified version - different number of levels | Inclusion criteria - sex, race, other  Facilitator(s)  1. Community leaders/sites - Sample size 1; sampling strategy judgmental;  Participant(s)  1. Men  Sample size 558; mean age 54.4;  Ethnicity black race;  Sampling strategy convenience;  Data collection method questionnaire; reliability check NR;  Outcome measure: screening practiced | Chi-square  Regression  Descriptive  other | Positive predictors - healthcare provider recommendations, insurance, other  Negative predictors - lack of healthcare provider recommendations, lack of access to healthcare providers/facilities  Overall view of model - NR |
| Mammography Facilities are Accessible, so Why is Utilization so Low?  (Mobley LR et al., 2009) | Determine the cause of behavior | USA | D: Breast cancer  T: Screening | Unclear  Cross-sectional  Other secondary | SEM by Bronfenbrenner  Modified version - different number of levels; different names/contents ; incorporates another model | Inclusion criteria - sex, age  Facilitator(s)  1. Database - Sample size 2;  Participant(s)  1. Women  Sample size 70129;  min age 65, max age 104;  Ethnicity - black, white, Latin, Asian, native and other; mixed vaccinated and not vaccinated; mixed screened and not screened;  Data collection method secondary data; reliability check NR;  Outcome measure: screening practiced | Regression  other | Positive predictors - positive influences and surroundings, insurance, medical status/practices, culture, other  Negative predictors - negative influences and surroundings, lack of access to healthcare providers/facilities, medical status/practices, culture, other  Overall view of model - NR |
| The Role of Religious Values in Decisions about Genetics and the Public's Health  (Modell SM, et al.; 2014) | Other | USA | D: Breast cancer  T: screening | Qualitative  Simple overview | SEM by other author  Not modified | Participant(s)  1. Book, journal article  Data collection method secondary data;  Outcome measure: other | Qualitative NR | Positive predictors - NR  Negative predictors - NR  Overall view of model - good |
| A Community-based Participatory Research Approach to Understanding Pap Testing Adherence among Vietnamese American Immigrants.  (Nguyen-Truong CKY, et al.; 2012) | Examine acceptance and non- acceptance of vaccination, screening or treatment | Urban  USA | D: Cervical cancer  T: screening | Quantitative  Descriptive study | SEM by other author  Modified version - different number of levels | Inclusion criteria - sex, age, race, health, other  Facilitator(s)  1. Community leaders/sites - Sample size 12; sampling strategy judgmental;  Participant(s)  1. Women  Sample size 211;  min age 12, max age 87, mean age 49.85;  Ethnicity - Asian; mixed screened and not screened;  Sampling strategy convenience;  Data collection method questionnaires; reliability check performed;  Outcome measure: screening practiced | Chi-square  Regression  descriptive | Positive predictors - positive influences and surroundings, healthcare provider recommendations, insurance, other  Negative predictors - negative influences and surroundings, lack of knowledge/awareness, culture, other  Overall view of model - NR |
| Applying the Social Ecological Model to Evaluate a Demonstration Colorectal Cancer Screening Program in Louisiana  (Nuss HJ, et al.; 2012) | Form or evaluate interventions | USA | D: Colon and Rectal Cancer  T: Screening | Unclear primary study | SEM by other author  Not modified | Inclusion criteria - age, SES, health  Facilitator(s)  1. Researchers/mediators - sampling strategy judgmental; recruitment other;  Participant(s)  1. Patients  Sample size 975;  mean age 55; male and female;  Ethnicity black, white, Asian, native and other race; mixed screened and not screened;  Sampling strategy judgmental;  Data collection method questionnaire and biophysical measurements; reliability check NR;  Outcome measure: screening practiced and screening not practiced | Chi-square, descriptive, t-test, other | Positive predictors - personal beliefs, access to healthcare providers/facilities, knowledge/awareness, law, other  Negative predictors - NR  Overall view of model - good |
| PCNA 19th Annual Symposium: Poster Abstracts.  (PCNA, 2013) | Explore views on vaccination, screening or disease/condition  Other | High income  Middle income  Multiple countries | D: Other  T: Screening | Unclear primary study | SEM not referenced | Inclusion criteria - other  Facilitator(s)  1. Healthcare professionals/facilities - sample size 5;  Participant(s)  1. Patients  Sample size 941;  mean age 50.4; male and female;  mixed screened and not screened;  Sampling strategy convenience;  Data collection method biophysical measurements; reliability check NR;  Outcome measure: other | Quantitative NR | Positive predictors -NR  Negative predictors - NR  Overall view of model - NR |
| Public Policy Approaches to the Prevention of Heart Disease and Stroke.  (Pearson AT, 2011) | Form or evaluate interventions | National  Community  USA | D: Other  T: Intervention: Other | Simple overview | SEM by Stokols  Modified version- incorporates another model | Outcome measure: screening practiced, non-risk behavior/treatment and other | NA | Positive predictors - policies/rules  Negative predictors - NR  Overall view of model - NR |
| Linkages Between Clinical Practices and Community Organizations for Prevention: A Literature Review and Environmental Scan.  (Porterfield SD, et al., 2012) | Form or evaluate interventions | Unclear | D: Other  T: Substance abuse/risk behavior  other | Other secondary study | SEM by McLeroy  Modified version- incorporates another model | Facilitator(s)  1. Databases - sample size 4;  2. Other - sample size 14; sampling strategy judgmental;  Participant(s)  1. Books, journal articles  Sample size 49;  Sampling strategy judgmental;  Data collection method secondary data; reliability check NR;  Outcome measure: screening practiced, non-risk behavior/treatment and other | NA | Positive predictors - NR  Negative predictors - NR  Overall view of model - NR |
| Social Work in the Emergency Department-implementation of a Domestic and Family Violence Screening Program  (Power C, et al.; 2011) | Form or evaluate interventions | Urban  Australia | D: Other  T: screening | Mixed methods  Other primary study | SEM by other author  Modified version - unclear | Inclusion criteria - sex, age  Facilitator(s)  1.Healthcare professionals/facility - sample size 109; sampling strategy convenience and judgmental;  Participant(s)  1. Women  not screened; sampling strategy convenience;  Data collection method questionnaire; reliability check NR;  Outcome measure: other  2. healthcare professionals  Sample size 40; male and female;  Data collection method questionnaire; reliability check NR;  Outcome measure: other | Thematic analysis  Descriptive | Positive predictors - positive influences and surroundings, knowledge/awareness, other  Negative predictors - negative influences and surroundings  Overall view of model - NR |
| Determinants of Mammography in Women with Intellectual Disabilities  (Wilkinson JE, et al.; 2011) | Form or evaluate interventions  Other | USA | D: Breast cancer  T: screening | Unclear primary study  Other secondary study | SEM by McLeroy  Modified version - different number of levels | Inclusion criteria - sex, age, health  Facilitator(s)  1. Databases  Sample size 1;  Participant(s)  1. Women  Sample size 2907;  mean age 57.70;  Data collection method secondary data; reliability check performed;  Outcome measure: screening practiced and not practiced | Chi –square  Regression  Correlation  other | Positive predictors - access to healthcare providers/facilities, medical status/practices, other  Negative predictors - medical status/practices, other  Overall view of model - NR |
| Social Ecological Predictors of Prostate-specific Antigen Blood Test and Digital Rectal Examination in Black American Men  (Woods VD, et al.; 2006) | Examine acceptance and non- acceptance of vaccination, screening or treatment | USA | D: Prostate Cancer  T: Screening | Quantitative  Cross-sectional  cohort | SEM by Stokols  Modified version - different names/contents | Inclusion criteria - sex, race, health  Facilitator(s)  1. Healthcare professionals/facilities  2. Community leaders/sites  Participant(s)  1. Men  Sample size 11;  max age 39, mean age 53; ethnicity - black; mixed screened and not screened;  Sample strategy convenience, judgmental;  Data collection method questionnaires; reliability check performed;  Outcome measure: screening practiced  2. Men  Sample size 265;  min age 40, max age 60, mean age 53;  Ethnicity - black; mixed screened and not screened;  Sample strategy convenience, judgmental;  Data collection method questionnaires; reliability check performed;  Outcome measure: screening practiced | Chi-square  Regression  Descriptive  Correlation  Z-test  tailed test  other | Positive predictors - personal beliefs, healthcare provider recommendations, knowledge/awareness, insurance, policies/rules, other  Negative predictors - negative personal beliefs, lack of access to healthcare providers/facilities, lack of knowledge/awareness, other  Overall view of model - NR |
| A Community-based Approach to Translational Research Addressing Breast Cancer Disparities.  (Yeary K, et al.; 2011) | Determine the cause of behavior  Form or evaluate interventions | Rural  USA | D: Breast cancer  T: Screening  other | Qualitative  Case study | SEM by McLeroy  Modified version - different names/contents | Inclusion criteria - sex, age ,race  Facilitator(s)  1. Community site/leaders - Sample size 6;  Participant(s)  1. Women  Sample size 56;  min age 40, mean age 69;  Ethnicity - black, white, mixed; Christian religion; mixed screened and not screened;  Sample strategy convenience;  Data collection method focus groups; reliability check NR;  Outcome measure: screening practiced and other | Constant comparison  Thematic analysis | Positive predictors - positive influences and surroundings, access to healthcare providers/facilities, knowledge/awareness, policies/rules, medical status/practices, other  Negative predictors - negative personal beliefs, lack of healthcare provider recommendations, lack of access to healthcare providers/facilities, lack of knowledge/awareness, lack of insurance, other  Overall view of model - Good |

NA = Not Applicable; NR = Not Reported; SES = Social Economic Status; D = Disease/condition; T = Treatment

Characteristics of study participants are indicated if the data was available in the study, if the data was of significance to the research

**Table S3: Characteristics of included TTI studies**

| **Study**  **(author(s), year)** | **Aim** | **Study Location** | **Target disease/ condition, treatment** | **Study Type** | **Model, Variables** | **Participants** | **Analysis** | **outcomes** |
| --- | --- | --- | --- | --- | --- | --- | --- | --- |
| Design of a School-based Randomized Trial to Reduce Smoking among 13 to 15-year olds, the X:IT Study.  (Andersen A, et al.; 2014) | Form or evaluate interventions  other | Denmark | D: Drug/substance abuse  T: Substance abuse/risk behavior | Quantitative  Randomized controlled trial | TTI by Petraitis & Flay  Modified version - different number of levels; different names/contents | Inclusion criteria - education  Facilitator(s)  1. Educational institute - sample size 94; sampling strategy stratified;  Participant(s)  1. Students  Sample size 4161; follow up was conducted;  male, female;  Ethnicity native, other; upper, middle, lower SES; secondary education;  Sampling strategy convenience;  Data collection method questionnaires; reliability check NR;  Outcome measure: non-risk behavior practiced/treatment and risk behavior practiced/no treatment  2. Parents  Follow up was conducted; male, female; upper, middle, lower SES;  Sampling strategy snowball;  Data collection method questionnaires; reliability check NR;  Outcome measure: non-risk behavior practiced/treatment and risk behavior practiced/no treatment  3. Teachers  follow up was conducted;  Data collection method questionnaires; reliability check NR;  Outcome measure: non-risk behavior practiced/treatment and risk behavior practiced/no treatment | descriptive | Positive predictors - NR  Negative predictors - NR  Overall view of model - NR |
| Motivational and Social Cognitive Predictors of Doping Intentions in Elite Sports: An Integrated Approach.  (Barkoukis V, et al.; 2013) | other | unclear | D: Drug/substance abuse  T: Other | Quantitative  Cross-sectional study | TTI by Petraitis & Flay  Modified version - different number of levels; incorporates another model | Inclusion criteria - SES, other  Facilitator(s)  1. Community leaders/sites - sampling strategy stratified;  Participant(s)  1. Other  Sample size 750;  mean age 25; male, female;  Data collection method questionnaire; reliability check performed;  Outcome measure: risk behavior practiced/no treatment | Regression  sobel test | Positive predictors - NR  Negative predictors - negative influences and surroundings, other  Overall view of model - NR |
| Illicit Use of Prescription Stimulants in a College Student Sample: A Theory-Guided Analysis  (Bavarian N, et al.; 2013) | Determine the cause of behavior  other | USA | D: Drug/substance abuse  T: Substance abuse/risk behavior | Quantitative  Cross-sectional study | TTI by Petraitis & Flay  Not modified | Inclusion criteria - age, education  Facilitator(s)  1. Education facility - Sample size 1; sampling strategy cluster;  Participant(s)  1. Students  Sample size 520;  min age 18; male, female;  Ethnicity white, Latin, Asian, other; degree education;  Sampling strategy convenience;  Data collection method questionnaires; reliability check NR;  Outcome measure: risk behavior practiced/no treatment | Regression  Descriptive | Positive predictors - positive influences and surroundings, other  Negative predictors - negative influences and surroundings, negative personal beliefs, other  Overall view of model - NR |
| An Exploratory Multilevel Analysis of Nonprescription Stimulant Use in a Sample of College Students  (Bavarian N, et al.; 2014) | other | USA | D: Drug/substance abuse  T: other | Quantitative  Cross-sectional  Other secondary study | TTI by Petraitis & Flay  Not modified | Inclusion criteria - education  Facilitator(s)  1. Educational institute - sample size 18;  Participant(s)  1. Students  Sample size 10220;  min age 18; male, female;  Ethnicity black, white, Latin, Asian, mixed, native, other; degree education;  Sampling strategy convenience, simple random;  Data collection method secondary data; reliability check performed;  Outcome measure: risk behavior practiced/no treatment | Chi-square, regression | Positive predictors - NR  Negative predictors - negative influences and surroundings, , medical status/practices, culture, other  Overall view of model - good |
| Using Structural Equation Modeling to Understand Prescription Stimulant Misuse: A Test of the Theory of Triadic Influence  (Bavarian N, et al.; 2014) | Determine the cause of behavior  Other | USA | D: Drug/substance abuse  T: Substance abuse/risk behavior | Quantitative  Cross-sectional study | TTI by Petraitis & Flay  Not modified | Inclusion criteria - age, education  Facilitator(s)  1. Educational institutions - sample size 1; sampling strategy simple random, cluster;  Participant(s)  1. Students  554 sample size;  min age 18; male, female;  Ethnicity black, white, Latin, Asian, other; degree education;  Sampling strategy convenience;  Data collection method questionnaire; reliability check performed;  Outcome measure: risk behavior practiced/no treatment | Descriptive, other | Positive predictors - positive influences and surroundings  Negative predictors - negative influences and surroundings, lack of access to healthcare providers/facilities  Overall view of model - NR |
| A Commentary on the Triadic Theory of Influence as a Guide for Adapting HIV Prevention Programs for New Contexts and Populations: The CHAMP-South Africa Story.  (Bell CC, et al.; 2007) | Form or evaluate interventions | Urban  Rural  South Africa | D: Other  T: Other | Unclear primary study | TTI by Petraitis & Flay  Not modified | Inclusion criteria - other  Facilitator(s)  1. Educational institute  Participant(s)  1. Other  Sample size 124;  Sampling strategy convenience, snowball;  reliability check performed  Outcome measure: other | Qualitative NR  Quantitative other | Positive predictors - NR  Negative predictors - NR  Overall view of model - better modified |
| The Critical Role of Nurturing Environments for Promoting Human Well-being.  (Biglan A, et al.; 2012) | other | NR | D: other  T: Substance abuse/risk behavior | systematic review | TTI by Petraitis & Flay  Modified version - different number of levels | Participant(s)  1. Books, journal articles  Data collection method secondary data; | NA | Positive predictors - access to healthcare providers/facilities, knowledge/awareness, policies/rules, other  Negative predictors - NR  Overall view of model - NR |
| Theories of How the School Environment Impacts on Student Health: Systematic Review and Synthesis.  (Bonell CP, et al.; 2013) | other | NR | D: Other  T: Other | Mixed methods  Systematic review | TTI by Petraitis & Flay  Modified version - incorporates another model | Inclusion criteria - age, health, education, other  Facilitator(s)  1. Database - sample size 16;  Participant(s)  1. Books, journal articles  Sample size 37;  Sampling strategy judgmental;  Data collection method secondary data;  Outcome measure: other | NA | Positive predictors - NR  Negative predictors - NR  Overall view of model - better modified |
| Family First: The Development of an Evidence-based Family Intervention for Increasing Participation in Psychiatric Clinical Care and Research in Depressed African American Adolescents  (Breland-Noble AM, et al; 2006) | Form or evaluate interventions | USA | D: Other  T: Other | Guideline | TTI by Petraitis & Flay  Modified version - incorporates another model | Inclusion criteria - race  Outcome measure: non-risk behavior practiced/treatment | NA | Positive predictors - NR  Negative predictors - NR  Overall view of model - NR |
| Psychological and Social Risk Factors in Adolescent Smoking Transitions: A Population-based Longitudinal Study.  (Bricker JB, et al.; 2009) | Determine the cause of behavior | USA | D: Drug/substance abuse  T: Other | Quantitative  Longitudinal  cohort | TTI by Petraitis & Flay  Modified version - different number of levels/constructs | Inclusion criteria - education, other  Facilitator(s)  1. Educational institute  Participant(s)  1. Adolescents  Sample size 4218; follow up was conducted; male, female;  Ethnicity white, NR race; secondary, primary education;  Data collection method questionnaires; reliability check performed;  Outcome measure: risk behavior practiced/no treatment  2. Parents  follow up not conducted; male, female;  Sampling strategy snowball;  Collection tool questionnaires; reliability check performed;  Outcome measure: non-risk behavior practiced/treatment and risk behavior practiced/no treatment | Quantitative other | Positive predictors - positive influences and surroundings  Negative predictors - negative influences and surroundings, negative personal beliefs  Overall view of model - good |
| Determinants of Tobacco Use among Korean Female Adolescents: Longitudinal Test of the Theory of Triadic Influence  (Chun J; 2014) | other | National  South Korea | D: Drug/substance abuse  T: Substance abuse/risk behavior | Longitudinal study  Other secondary study | TTI by Petraitis & Flay  Not modified | Inclusion criteria - sex, education  Facilitator(s)  1. Databases  Participant(s)  1. Adolescents  Sample size 1594; follow up was conducted; female;  secondary education;  Sampling strategy stratified, cluster; consent NR;  Data collection method questionnaire, secondary data; reliability check performed;  Outcome measure: risk behavior practiced/no treatment | Chi-square  Regression  Descriptive statistics  other | Positive predictors - positive influences and surroundings, personal beliefs  Negative predictors - negative influences and surroundings, culture  Overall view of model - good |
| Gender Differences in Factors Influencing Smoking, Drinking, and Their Co-occurrence among Adolescents in South Korea.  (Chun J & Chung IJ; 2013) | Determine the cause of behavior | South Korea | D: Drug/substance abuse  T: Substance abuse/risk behavior | Unclear primary study  Other secondary study | TTI by Petraitis & Flay  Modified version - different number of levels | Inclusion criteria - education  Facilitator(s)  1. Databases - Sample size 1; sampling strategy judgmental;  Participant(s)  1. Students  Sample size 3188 follow up was conducted;  mean age 15; male and female;  Ethnicity native; secondary education;  Sampling strategy stratified, cluster;  Data collection method questionnaires, secondary data; reliability check performed;  Outcome measure: risk behavior practiced/no treatment | Chi-square  Regression  Descriptive statistics | Positive predictors - positive influences and surroundings, personal beliefs, policies/rules  Negative predictors - negative influences and surroundings, negative personal beliefs, culture, other  Overall view of model - NR |
| The Grady Nia Project: A Culturally Competent Intervention for Low-income, Abused, and Suicidal African American Women  (Davis SP; 2009) | Form or evaluate interventions | USA | D: other  T: Substance abuse/risk behavior | guideline | TTI by Petraitis & Flay  Modified version - different number of levels/constructs; incorporates another model | Inclusion criteria - sex, race  NA | NA | Positive predictors - positive influences and surroundings, personal beliefs, access to healthcare providers/facilities, culture  Negative predictors - NR  Overall view of model - NR |
| Determinants of Adolescent Bicycle Use for Transportation and Snacking Behavior  (de Bruijn G, et al.; 2005) | Determine the cause of behavior  other | Netherlands | D: Other  T: Other | Quantitative  Cross-sectional study | TTI by Petraitis & Flay  Modified version - different number of levels/constructs; incorporates another model | Inclusion criteria - education  Facilitator(s)  1. Educational institute - sample size 75; sampling strategy simple random;  Participant(s)  1. Students  Sample size 3859;  mean age 14.8; male, female;  Ethnicity native, other; secondary, degree education;  Data collection tool questionnaires; reliability check performed;  Outcome measure: non-risk behavior practiced/treatment and risk behavior practiced/no treatment | Regression, correlation | Positive predictors - culture, other  Negative predictors - culture, other  Overall view of model - NR |
| Predictors of Binge Drinking in Adolescents: Ultimate and Distal Factors - a Representative Study  (Donath C, et al.; 2012) | Determine the cause of behavior | Urban  Rural  Germany | D: Drug/substance abuse  T': Other | Quantitative  Cross-sectional study | TTI by Petraitis & Flay  Modified version - different number of levels/constructs | Inclusion criteria - education  Facilitator(s)  1. Educational institute - sample size 2131; sampling strategy stratified;  Participant(s)  1. Adolescents  Sample size 44610;  mean age 15.3; male, female;  secondary education;  Sampling strategy convenience;  Data collection method questionnaire; reliability check performed;  Outcome measure: risk behavior/no treatment | Chi-square, regression, correlation, other | Positive predictors - personal beliefs  Negative predictors - negative influences and surroundings, negative personal beliefs, other  Overall view of model - better modified |
| Targets for Primary Prevention: Cultural, Social and Intrapersonal Factors Associated with Co-occurring Health-related Behaviors.  (Dusseldorp E, et al.; 2014) | Form or evaluate interventions | National  Netherlands | D: Other  T: Other | Cross-sectional study | TTI by Petraitis & Flay  Modified version - different number of levels/constructs | Inclusion criteria - other  Facilitator  1. Database - sample size 1;  Participants  1. other  Sample size 3497;  min age 19, max age 40, mean age 30.64; male, female;  Ethnicity native, other; degree, secondary, primary education; married/dating, single, divorced;  Sampling strategy stratified;  Data collection method questionnaire and interviews; reliability check NR;  Outcome measure: non-risk behavior practiced/treatment and risk behavior practiced/no treatment | Regression, correlation | Positive predictors - positive influences and surroundings, personal beliefs  Negative predictors - NR  Overall view of model - good |
| Effects of 2 Prevention Programs on High-risk Behaviors among African American Youth: A Randomized Trial.  (Flay BR, et al.; 2004) | Form or evaluate interventions | USA | D: other  T: Substance abuse/risk behavior | Randomized controlled trial  cohort | TTI by Petraitis & Flay  Modified version - unclear | Inclusion criteria - race, SES, education, other  Facilitator(s)  1. Education institution - sample size 12; sampling strategy judgmental, stratified, other;  Participant(s)  1. Students  Sample size 664; follow up was conducted;  mean age 10.8; male, female;  Ethnicity black ,Latin, NR; primary education;  Sampling strategy convenience;  Data collection method questionnaires; reliability check performed;  Outcome measure: risk behavior practiced/no treatment | Regression  tailed test  other | Positive predictors - positive influences and surroundings, personal beliefs, other  Negative predictors - other  Overall view of model - NR |
| A Culturally-informed Approach to Trauma, Suicidal Behavior, and Overt Aggression in African American Adolescents  (Graves KN, et al.; 2010) | Determine the cause of behavior | USA | D: Other  T: Other | Simple overview | TTI by Petraitis & Flay  Modified version - different number of levels | Inclusion criteria - age, race  Participant(s)  1. Book, journal articles  Data collection method secondary data;  Outcome measure: other; | NA | Positive predictors - positive influences and surroundings, personal beliefs  Negative predictors - negative influences and surroundings, negative personal beliefs, other  Overall view of model - NR |
| Influences Affecting Adolescent Smoking Behavior in China.  (Grenard JL, et al.; 2006) | other | China | D: Drug/substance abuse  T: other | Quantitative  Longitudinal  Other secondary study | TTI by Petraitis & Flay  Not modified | Inclusion criteria - education  Facilitator(s)  1. Educational institute - sample size 147; follow up was conducted; Sampling strategy stratified;  Participant(s)  1. Students  Sample size 11583; follow up was conducted; male, female;  Ethnicity native, other, NR; secondary, primary education;  Sampling strategy convenience;  Data collection method secondary data; reliability check performed;  Outcome measure: risk behavior practiced/no treatment  2. Parents  follow up was conducted;  Sampling strategy snowball;  Data collection method secondary data; reliability check performed  Outcome measure: NR | Chi-square  t-test  other | Positive predictors - NR  Negative predictors - negative influences and surroundings, other  Overall view of model - NR |
| Concurrent Predictors of Cigarette and Alcohol Use among U.S. and Russian Adolescents  (Gunning M, et al.; 2009) | other | USA, Russia | D: Drug/substance abuse  T: other | Quantitative  Cross-sectional study | TTI by Petraitis & Flay  Modified version - different number of levels | Inclusion criteria - sex, age, race, education  Facilitator(s)  1. Education institutions - sample size 10; sampling strategy convenience;  Participant(s)  1. Students  Sample size 365;  mean age 15.65; male, female;  Ethnicity white, Asian, mixed, other; secondary education;  Data collection method questionnaires; reliability check performed;  Outcome measure: risk behavior practiced/no treatment  2. students  Sample size 965;  mean age 15.14; male, female;  Ethnicity black, white, Latin, Asian, mixed, native, other; secondary education;  Data collection method questionnaires; reliability check conducted;  Outcome measure: risk behavior practiced/no treatment | Regression, correlation, tailed test, other | Positive predictors - personal beliefs  Negative predictors - negative influences and surroundings, other  Overall view of model - good |
| A Multilevel-based Study of School Policy for Tobacco Control in Relation to Cigarette Smoking among Children in Elementary Schools: Gender Differences.  (Huang HL, et al.; 2010) | Explore views on vaccination, screening or disease/condition  other | Urban  Rural  Taiwan | D: Drug/substance abuse  T: Substance abuse/risk behavior | Cross-sectional study | TTI by Petraitis & Flay  Modified version - different number of levels | Inclusion criteria - education  Facilitator(s)  1. Education institution - sample size 26; sampling strategy cluster;  Participant(s)  1. Students  Sample size 2350;  min age 10.9; male, female;  primary education;  Sampling strategy convenience;  Data collection method questionnaires; reliability check NR;  Outcome measure: non-risk behavior practiced/treatment and risk behavior practiced/no treatment;  2. Parents  Sampling strategy snowball;  Data collection method questionnaires; reliability check NR;  Outcome measure: NR  3. Teachers  Sample size 52;  Sampling strategy judgmental;  Data collection method questionnaires; reliability check NR;  Outcome measure: other | regression | Positive predictors - knowledge/awareness, policies/rules  Negative predictors - negative influences and surroundings, lack of knowledge/awareness, culture, other  Overall view of model - NR |
| School-level Contextual Factors Associated with Betel Quid Chewing among Schoolchildren in Taiwan.  (Huang HL, et al.; 2009) | Determine the cause of behavior | Taiwan | D: Drug/substance abuse  T: Substance abuse/risk behavior | Quantitative  Cross-sectional study  Other secondary study | TTI by Petraitis & Flay  Modified version - different number of levels/constructs | Inclusion criteria - race, other  Facilitator(s)  1. Educational institute - sample size 13; sampling strategy simple random;  Participant(s)  1. Students  Sample size 1585;  mean age 8.9; male, female;  upper, middle, lower SES; primary education;  Sampling strategy convenience;  Data collection method questionnaires, secondary data; reliability check unclear;  Outcome measure: non-risk behavior practiced/treatment and risk behavior practiced/no treatment  2. Parents  male, female; upper, middle, lower SES; degree, secondary, primary;  Sampling strategy snowball;  Data collection method questionnaires, secondary data; reliability check unclear;  Outcome measure: non-risk behavior practiced/treatment and risk behavior practiced/no treatment | regression | Positive predictors - NR  Negative predictors - negative influences and surroundings, culture, other  Overall view of model - NR |
| Starting to Smoke: A Qualitative Study of the Experiences of Australian Indigenous Youth.  (Johnston V, et al.; 2012) | Determine the cause of behavior | Urban  Rural  Australia | D: Drug/substance abuse  T: Substance abuse/risk behavior | Qualitative  Unclear primary study | TTI by Petraitis & Flay  Not modified | Inclusion criteria - age, race  Facilitator(s)  1. Researchers, mediators - sample size 6  Participant(s)  1. Youth  Sample size 65;  min age 13, max age 20, mean age 15.6; male, female;  Ethnicity native, other;  Sampling strategy convenience, cluster;  Data collection method interviews, focus groups, other; reliability check NR;  Outcome measure: risk behavior practiced/no treatment | Constant comparison  Thematic analysis | Positive predictors - positive influences and surroundings, policies/rules, other  Negative predictors - negative influences and surroundings, negative personal beliefs, policies/rules, other  Overall view of model - NR |
| Suicidal, Abused African American Women's Response to a Culturally Informed Intervention  (Kaslow NJ, et al.; 2010) | Form or evaluate interventions | Urban  USA | D: Other  T: Substance abuse/risk behavior | Randomized controlled trial | TTI by Petraitis & Flay  Modified version - different number of levels/constructs | Inclusion criteria - sex, race, health  Facilitator(s)  1. Healthcare professionals/facility - sample size 1;  Participant(s)  1. Women  Sample size 131; follow up was conducted;  min age 18; max age 64;  Ethnicity black race; lower SES; married/dating;  Sampling strategy simple random;  Data collection method interviews; reliability check performed;  Outcome measure: risk behavior/no treatment | Chi-square, regression, other | Positive predictors - positive influences and surroundings, culture  Negative predictors - NR  Overall view of model - better modified |
| Cultural, Social and Intrapersonal Factors Associated with Clusters of Co-occurring Health-related Behaviors among Adolescents.  (Klein Velderman M, et al.; 2015) | Determine the cause of behavior  other | National  Netherlands | D: Other  T: Other | Quantitative  Cross-sectional study | TTI by Petraitis & Flay  Modified version - different number of levels | Inclusion criteria - sex, age, education  Participant(s)  1. Adolescents  Sample size 898;  min age 12, max age 18; male, female;  Data collection method questionnaire, interview; reliability check performed;  Outcome measure: risk behavior practiced/no treatment; | Regression, descriptive, correlation | Positive predictors - positive influences and surroundings, personal beliefs  Negative predictors - other  Overall view of model - NR |
| Brief Report: The Adaptation of Project Northland for Urban Youth.  (Komro KA, et al.; 2004) | Form or evaluate interventions | Urban  USA | D: Drug/substance abuse  T: Substance abuse/risk behavior | Randomized controlled trial  Simple overview | TTI by Petraitis & Flay  Modified version - different number of levels/constructs; incorporates another model | Inclusion criteria - education  Facilitator(s)  1. Educational institutes - sample size 61;  Participant(s)  1.Students  Sample size 4164, follow up conducted;  Ethnicity black, white, Latin, Asian, mixed, native; primary education;  Data collection method observation, questionnaire, focus groups; reliability check NR;  Outcome measure: risk behavior practiced/no treatment  2. Parents  Data collection method questionnaire, interviews; reliability check NR;  Outcome measure: other  3. Community leaders  Data collection method questionnaire; reliability check NR;  Outcome measure: NR | NR | Positive predictors - NR  Negative predictors - NR  Overall view of model - NR |
| Participation in a Sigmoidoscopic Colorectal Cancer Screening Program: A Pilot Study  (Kremers SP, et al.; 2000) | Examine acceptance and non- acceptance of vaccination, screening or treatment | Netherlands | D: Colon and Rectal Cancer  T: Screening | Quantitative  Other primary study | TTI by Petraitis & Flay  Modified version - different number of levels | Inclusion criteria - age, health  Facilitator(s)  1. Healthcare professionals/facilities - sample size 2;  Participant(s)  1. Patients  Sample size 200;  min age 50, max age 60, mean age 55.4; male and female;  degree, secondary, primary education; mixed screened and not screened;  Sampling strategy convenience, judgmental;  Data collection method questionnaires, biophysical tests; reliability check performed;  Outcome measure: screening practiced | Chi-square, regression, descriptive, z-test | Positive predictors - positive influences and surroundings, knowledge/awareness, medical status/practices  Negative predictors - negative personal beliefs, medical status/practices, other  Overall view of model - better modified |
| Description of an Efficacious Behavioral Peer-driven Intervention to Reduce Racial/Ethnic Disparities in AIDS Clinical Trials.  (Leonard NR, et al.; 2013) | Form or evaluate interventions | USA | D: Other  T: screening | Guideline | TTI by Petraitis & Flay  Modified version - different number of levels; different names/contents ; incorporates another model | Inclusion criteria - race  Outcome measure: screening practiced | NA | Positive predictors - access to healthcare providers/facilities, other  Negative predictors - other  Overall view of model - NR |
| Health-promoting and Health-risk Behaviors: Theory-driven Analyses of Multiple Health Behavior Change in Three International Samples  (Lippke S, et al.; 2012) | other | National  USA, Germany | D: Other  T: Other | Quantitative  Cross -sectional study | TTI by Petraitis & Flay  Modified version - unclear | Inclusion criteria - health, other  Participant(s)  1. Adults  Sample size 3519;  min age 18, max age 91, mean age 46.31; male, female;  degree, secondary, primary education;  Sampling strategy simple random;  Data collection method interviews; reliability check not performed;  Outcome measure: non-risk behavior practiced/treatment and risk behavior practiced/no treatment  2. Other  Sample size 961;  min age 15, max age 81, mean age 39.21; male, female;  degree, secondary, primary education;  Sampling strategy convenience;  Data collection method questionnaires; reliability check not performed;  Outcome measure: non-risk behavior practiced/treatment and risk behavior practiced/no treatment  3. Adults  Sample size 310;  min age 18, max age 75, mean age 43.52; male, female;  degree, secondary education;  Sampling strategy convenience, snowball;  Data collection method questionnaires; reliability check not performed;  Outcome measure: non-risk behavior practiced/treatment and risk behavior practiced/no treatment | Correlation, other | Positive predictors - other  Negative predictors - other  Overall view of model - NR |
| Predicting Self-initiated Marijuana Use Cessation among Youth at Continuation High Schools.  (Little MA, et al.; 2013) | Determine the cause of behavior | USA | D: Drug/substance abuse  T: Substance abuse/risk behavior | Quantitative  Longitudinal study | TTI by Petraitis & Flay  Modified version - different number of levels | Inclusion criteria - education  Facilitator(s)  1. Educational institutions - sample size 24; follow up was conducted; sampling strategy convenience;  Participant(s)  1. Students  Sample size 522; follow up was conducted;  min age 14, max age 20, mean age 16.7; male and female;  Ethnicity black, white, Latin, Asian, mixed, native, other; secondary education;  Sampling strategy convenience;  Data collection method questionnaire; reliability check performed;  Outcome measure: non-risk behavior practiced/treatment | Chi-square, regression, t-test, tailed test, other | Positive predictors - positive influences and surroundings, personal beliefs  Negative predictors - negative influences and surroundings, negative personal beliefs  Overall view of model - NR |
| The Development and Implementation of Theory-driven Programs Capable of Addressing Poverty-impacted Children's Health, Mental Health, and Prevention Needs: CHAMP and CHAMP+, Evidence-informed, Family-based Interventions to Address HIV Risk and Care.  (McKay MM, et al.; 2014) | Form or evaluate interventions | High income  Middle income  Multiple countries | D: Other  T: Other | Simple overview | TTI by Petraitis & Flay  Modified version - different number of levels | Inclusion criteria - race  NA | NA | Positive predictors - NR  Negative predictors - NR  Overall view of model - good |
| A Multilevel Analysis Examining the Association Between School-based Smoking Policies, Prevention Programs and Youth Smoking Behavior: Evaluating a Provincial Tobacco Control Strategy.  (Murnaghan DA, et al.; 2008) | Determine the cause of behavior | Canada | D: Drug/substance abuse  T: Substance abuse/risk behavior | Quantitative  Cross-sectional study | TTI by Petraitis & Flay  Modified version - different number of levels | Inclusion criteria - education  Facilitator(s)  1. Education institution - sample size 10; no exclusions; sampling strategy judgmental;  Participant(s)  1. Students  Sample size 4732; male, female;  Data collection method questionnaires; reliability check NR;  Outcome measure: non-risk behavior practiced/treatment and risk behavior practiced/no treatment; | Chi-square, regression, descriptive | Positive predictors - positive influences and surroundings, knowledge/awareness, policies/rules  Negative predictors - negative influences and surroundings, policies/rules  Overall view of model - NR |
| Cultural and Social Influences on Food Consumption in Dutch Residents of Turkish and Moroccan Origin: A Qualitative Study.  (Nicolaou M, et al.; 2009) | Determine the cause of behavior  Form or evaluate interventions | Netherlands | D: Other  T: Other | Qualitative  Unclear primary study | TTI by Petraitis & Flay  Modified version - different number of levels/constructs | Inclusion criteria - sex, age ,race  Facilitator(s)  1. Community leaders/sites - sample size 6;  Participant(s)  1. Other  Sample size 79; male and female;  Ethnicity Arab; degree, secondary, primary education; Muslim religion;  Sampling strategy convenience;  Data collection method focus group; reliability check NR;  Outcome measure: other | Framework analysis | Positive predictors - culture, other  Negative predictors - negative influences and surroundings, negative personal beliefs  Overall view of model - better modified |
| Adolescent Psychological and Social Predictors of Young Adult Smoking Acquisition and Cessation: A 10-Year Longitudinal Study.  (Otten R, et al.; 2011) | Determine the cause of behavior | USA | D: Drug/substance abuse  T: other | Longitudinal  cohort | TTI by Petraitis & Flay  Modified version - different number of levels | Inclusion criteria - age, health  Participant(s)  1.Adolesents  Sample size 2970; follow up was conducted; male, female;  Data collection method questionnaires; reliability check NR;  Outcome measure: non-risk behavior practiced/treatment and risk behavior practiced/no treatment; | Chi-square, other | Positive predictors - positive influences and surroundings  Negative predictors - negative influences and surroundings, negative personal beliefs  Overall view of model - good |
| Project Northland: Long-term Outcomes of Community Action to Reduce Adolescent Alcohol Use.  (Perry CL, et al.; 2002) | Form or evaluate interventions | Rural  USA | D: Drug/substance abuse  T: Substance abuse/risk behavior | Randomized controlled trial | TTI by Petraitis & Flay  Modified version - unclear | Inclusion criteria - SES, education  Facilitator(s)  1. Educational institutes - sample size 24; exclusions were made;  Participant(s)  1. Students  Sample size 3151; follow up was conducted;  middle and low SES; primary education;  Data collection method questionnaire; reliability check performed;  Outcome measure: risk behavior practiced/no treatment  2. Parents  Sample size 2048 in 1996; 1793 in 1998; follow up was conducted;  middle and low SES;  Data collection tool questionnaire; reliability check conducted;  Outcome measure: risk behavior practiced/no treatment | Regression  other | Positive predictors - positive influences and surroundings, other  Negative predictors - negative influences and surroundings, other  Overall view of model - NR |
| A Review of Similarities between Domain-specific Determinants of Four Health Behaviors among Adolescents.  (Peters LWH, et al.; 2009) | Determine the cause of behavior  Form or evaluate interventions | Netherlands | D: other  T: Substance abuse/risk behavior  other | Systematic review | TTI by Petraitis & Flay  Not modified | Inclusion criteria - age, other  Facilitator(s)  1. Databases - Sample size 2; sampling strategy judgmental  Participant(s)  1. Book, journal articles  Sample size 87;  Sampling strategy judgmental;  Data collection method secondary data;  Outcome measure: other | Qualitative other | Positive predictors - positive influences and surroundings, personal beliefs  Negative predictors - negative influences and surroundings, negative personal beliefs, other  Overall view of model - NR |
| Effects of Transfer-oriented Curriculum on Multiple Behaviors in the Netherlands.  (Peters LWH, et al.; 2015) | other | Netherlands | D: other  T: Substance abuse/risk behavior  other | Cross-sectional study  Other primary study | TTI by Petraitis & Flay  Modified version - unclear | Inclusion criteria - race, education  Facilitator(s)  1. Educational institute - sample size 23; sampling strategy simple random;  Participant(s)  1. Students  follow up was conducted;  mean age 13.5; male, female;  Ethnicity mixed, native; secondary education;  Sampling strategy convenience;  Data collection method questionnaires; reliability check performed;  Outcome measure: other | Regression  t-test  other | Positive predictors - other  Negative predictors - NR  Overall view of model - NR |
| Sexual Violence and Youth in South Africa: The Need for Community-based Prevention Interventions  (Petersen I, et al.; 2005) | Determine the cause of behavior | Rural  South Africa | D: other  T: Substance abuse/risk behavior | Qualitative  Case-study | TTI by Petraitis & Flay  Modified version - different names/contents | Inclusion criteria - age  Facilitator(s)  1. Educational institute - sample size 1;  Participant(s)  1. Adolescents  Sample size 10 and 10; follow up was conducted;  min age 13, max age 16; male, female;  Ethnicity black;  Sampling strategy convenience;  Data collection method interview, focus groups; reliability check NR;  Outcome measure: risk behavior practiced/no treatment | Constant comparison  Thematic analysis  other | Positive predictors - NR  Negative predictors - negative influences and surroundings, negative personal beliefs, culture, other  Overall view of model - good |
| Youth Culture and Smoking: Integrating Social Group Processes and Individual Cognitive Processes in a Model of Health-Related Behaviors.  (Schofield PE, et al.; 2003) | other | Australia | D: Drug/substance abuse  T: other | cohort | TTI by Petraitis & Flay  Modified version - different number of levels/constructs; incorporates another model | Inclusion criteria - other  Facilitator(s)  1. Educational institute - sample size 93; sampling strategy stratified;  Participant(s)  1. Adults  Sample size 1584, 1423, 1379; follow up was conducted; male, female;  secondary education;  Sampling strategy simple random;  Data collection method questionnaires; reliability check performed;  Outcome measure: non-risk behavior practiced/treatment and risk behavior practiced/no treatment | Chi-square, z-test, other | Positive predictors - NR  Negative predictors - negative influences and surroundings, other  Overall view of model - better modified |
| Maternal Expectations, Mother-Child Connectedness, and Adolescent Sexual Debut.  (Sieving RE, et al.; 2000) | other | USA | D: Other  T: Other | Longitudinal study | TTI by Petraitis & Flay  Modified version - different number of levels/constructs | Inclusion criteria - education, other  Facilitator(s)  1. Educational institute - sampling strategy stratified;  Participant(s)  1. Adolescents  Sample size 3322;  follow up was conducted; male, female;  Ethnicity black ,white, Latin, other race; secondary education;  Sampling strategy interviews; reliability check performed;  Outcome measure: risk behavior practiced/no treatment and other  2. Parents  follow up was conducted; female;  Sampling strategy interviews; reliability check performed;  Outcome measure: other | Regression  Correlation  other | Positive predictors - positive influences and surroundings  Negative predictors - other  Overall view of model - NR |
| Community Perceptions of Adequate Levels and Reasons for Skin Protection.  (Stanton WR, et al.; 2005) | Explore views on vaccination, screening or disease/condition  Determine the cause of behavior | Australia | D: Melanoma  T: other | Quantitative  Unclear primary study | TTI by Petraitis & Flay  Modified version - different number of levels/constructs | Inclusion criteria - age  Participant(s)  1. Adults  Sample size 36000;  min age 18; male, female;  sampling strategy simple random;  Data collection tool questionnaire; reliability check performed;  Outcome measure: non-risk behavior practiced/treatment and risk behavior practiced/no treatment; | ANOVA | Positive predictors - positive influences and surroundings, personal beliefs, healthcare provider recommendations, medical status/practices  Negative predictors - negative influences and surroundings, negative personal beliefs, lack of knowledge/awareness, medical status/practices, other  Overall view of model - NR |
| The One-year Prospective Prediction of Substance Abuse and Dependence among High-risk Adolescents.  (Sussman S, et al.; 2000) | Determine the cause of behavior | USA | D: Drug/substance abuse  T: Substance abuse/risk behavior | Quantitative  Longitudinal study | TTI by Petraitis & Flay  Modified version - different number of levels/constructs | Inclusion criteria - sex, race, SES  Facilitator(s)  1. Educational institutions - Sample size 21;  Participant(s)  1. Students  Sample size 702; follow up was conducted;  mean age 16.8; male, female;  Ethnicity black, white, Latin; secondary education;  Data collection method questionnaires; reliability check performed;  Outcome measure: other | Regression | Positive predictors - NR  Negative predictors - negative influences and surroundings, negative personal beliefs, other  Overall view of model - NR |
| Concurrent Predictors of Drug Use Consequences among U.S. and Russian Adolescents.  (Sussman S, et al.; 2009) | other | USA, Russia | D: Drug/substance abuse  T: other | Quantitative  Cross-sectional study | TTI by Petraitis & Flay  Modified version - different number of levels/constructs | Inclusion criteria - sex, age, race, education  Facilitator(s)  1. Education institution - sample size 10; sampling strategy convenience;  Participant(s)  1. students  Sample size 365;  mean age 15.65; male, female;  Ethnicity white, Asian, mixed, other; secondary education;  Data collection method questionnaires; reliability check performed;  Outcome measure: risk behavior practiced/no treatment  2. Student(s)  Sample size 965;  mean age 15.14; male, female;  Ethnicity black, white, Latin, Asian, mixed, native, other; secondary education;  Data collection method questionnaires; reliability check performed;  Outcome measure: risk behavior practiced/no treatment | Regression, tailed test  other | Positive predictors - personal beliefs  Negative predictors - negative influences and surroundings, negative personal beliefs  Overall view of model - NR |
| Prospective Predictors of Technology-Based Sexual Coercion by College Males.  (Thompson MP & Morrison DJ; 2013) | Determine the cause of behavior  other | unclear | D: Other  T: Other | Unclear primary study | TTI by Petraitis & Flay  Modified version - different number of levels/constructs | Inclusion criteria - sex, age, education  Facilitator(s)  1. Education institute - sample size 1;  Participant(s)  1.Students  Sample size 571, follow up was conducted; male;  degree education; religion none and other;  sampling strategy convenience;  Data collection method questionnaires; reliability check performed;  Outcome measure: risk behavior practiced/no treatment | Regression, correlation | Positive predictors - other  Negative predictors - negative influences and surroundings, negative personal beliefs, other  Overall view of model - good |
| Obesity Prevention in Low Socioeconomic Status Urban African-American Adolescents: Study Design and Preliminary Findings of the Health-Kids Study.  (Wang Y, et al.; 2006) | Form or evaluate interventions | USA | D: Other  T: Other | Mixed methods  Cross-sectional  cohort | TTI by Petraitis & Flay  Modified version - other | Inclusion criteria - race, education, SES  Facilitator(s)  1. Educational institutions - sample size 4, follow up conducted; sampling strategy judgmental;  Participant(s)  1. Students  Sample size 450; follow up was conducted; male and female;  Ethnicity black, white, Latin; primary education;  Sampling strategy convince, judgmental, simple random;  Data collection method focus groups; reliability check performed;  Outcome measure: other  2. Parents  Sample size 230;  Sampling strategy judgmental, snowball, simple random;  Data collection method questionnaires, focus groups; reliability check performed;  Outcome measure: other  3. Other  Sampling strategy judgmental, simple random;  Data collection method observation, focus groups; reliability check performed;  Outcome measure: other | Thematic analysis  Quantitative other | Positive predictors - knowledge/awareness, other  Negative predictors - negative influences and surroundings, lack of access to healthcare providers/facilities, other  Overall view of model - good |
| Developmental Trajectories of Cigarette Use and Associations with Multilayered Risk Factors among Chinese Adolescents.  (Xie B, et al.; 2013) | Other | Urban  Rural  China | D: Drug/substance abuse  T: other | Quantitative  Longitudinal  Other secondary study | TTI by Petraitis & Flay  Modified version - different number of levels/constructs | Inclusion criteria - education  Facilitator(s)  1. Educational institute - sample size 22;  Participant(s)  1. Adolescents  Sample size 3521; follow up was conducted;  min age 12, max age 15; male, female;  primary education;  Sampling strategy simple random;  Data collection method secondary data; reliability check performed;  Outcome measure: non-risk behavior practiced/treatment and risk behavior practiced/no treatment | Descriptive, other | Positive predictors - positive influences and surroundings, policies/rules  Negative predictors - negative influences and surroundings, negative personal beliefs  Overall view of model - NR |

NA = Not Applicable; NR = Not Reported; SES = Social Economic Status; D = Disease/Condition; T = Treatment

Characteristics of study participants are indicated if the data was available in the study, if the data was of significance to the research

**Table S4: Quality assessment of studies using the SEM**

| **Study**  **(author(s), year)** | **Clarity of CRQ(s)/ hypothesis** | **Clarity of data collection methods** | **Clarity of sampling plan** | **Clarity of sampling size** | **Clarity of analysis method** | **Clarity of conclusions** | **Clarity of limitations** | **Overall quality of study** |
| --- | --- | --- | --- | --- | --- | --- | --- | --- |
| Factors Contributing to Filipinos' Resistance to Preventive Screening.  (Atassi K, et al.; 2010) | 1 | 1 | 0 | 1 | 0 | 1 | 0 | 4 |
| Factors Predicting BCG Immunization Status in Northern Nigeria: A Behavioral-ecological Perspective.  (Babalola S & Lawan U; 2009) | 1 | 2 | 1 | 1 | 1 | 1 | 0 | 7 |
| Enhancing Benefits or Increasing Harms: Community Responses for HIV among Men Who have Sex with Men, Transgender Women, Female Sex Workers, and People Who Inject Drugs.  (Baral S, et al.; 2014) | 1 | 2 | 2 | 2 | 0 | 1 | 0 | 8 |
| Understanding the Interplay of Factors Informing Vaccination Behavior in Three Canadian Provinces.  (Boerner F, et al; 2013) | 2 | 2 | 2 | 2 | 1 | 1 | 2 | 12 |
| Theorizing Social Context: Rethinking Behavioral Theory  (Burke NJ, et al.; 2009) | 0 | 0 | 0 | 0 | 0 | 1 | 0 | 1 |
| Willingness to Participate in HIV Vaccine Trials among Men Who have Sex with Men in Chennai and Mumbai, India: A Social Ecological Approach  (Chakrapani V, et al.; 2012) | 1 | 2 | 2 | 2 | 1 | 1 | 2 | 11 |
| American Heart Association Childhood Obesity Research Summit Report.  (Daniels RS, et al.; 2009) | 2 | 0 | 0 | 0 | 0 | 1 | 2 | 5 |
| Perspectives of African American, Amish, Appalachian and Latina women on Breast and Cervical Cancer Screening: Implications for Cultural Competence.  (Documét PI, et al.; 2008) | 1 | 2 | 1 | 2 | 1 | 1 | 2 | 10 |
| Increasing Use of Mammography among Older, Rural African American Women: Results from a Community Trial.  (Earp JA et al, 2002) | 0 | 2 | 2 | 2 | 1 | 1 | 2 | 10 |
| Beyond Effectiveness: Evaluating the Public Health Impact of the WISEWOMAN Program.  (Farris RP, et al.; 2007) | 2 | 2 | 0 | 2 | 1 | 1 | 2 | 10 |
| Barriers and Facilitators to HPV Vaccination of Young Women in High-income Countries: A Qualitative Systematic Review and Evidence Synthesis.  (Ferrer HB, et al.; 2014) | 2 | 2 | 2 | 2 | 1 | 1 | 2 | 12 |
| Behavioral Science Research in the Prevention of Diabetes : Status and Opportunities.  (Fisher EB, et al.; 2002) | 2 | 0 | 0 | 0 | 0 | 1 | 0 | 3 |
| Applying core principles to the design and evaluation of the 'Take Charge. Take the Test' campaign: what worked and lessons learned.  (Fraze JL et al.., 2009) | 0 | 1 | 0 | 0 | 1 | 1 | 1 | 4 |
| "I Connect with the Ringleader:" Health Professionals' Perspectives on Promoting the Sexual Health of Adolescent Males.  (Garcia MC, et al.; 2014) | 2 | 2 | 2 | 2 | 1 | 1 | 0 | 10 |
| Health Disparities in Colorectal Cancer Screening in the United States: An Application of the Social Ecological Model  (Greene DM; 2011) | 0 | 0 | 0 | 0 | 0 | 1 | 1 | 2 |
| Social Context and Drivers of Intimate Partner Violence in Rural Kenya: Implications for the Health of Pregnant Women.  (Hatcher AM, et al.; 2013) | 1 | 2 | 1 | 2 | 1 | 1 | 2 | 10 |
| Using Technology to Expedite Screening and Intervention for Domestic Abuse and Neglect.  (Hawkins JW, et al.; 2009) | 1 | 2 | 2 | 0 | 0 | 1 | 2 | 8 |
| Lay Health Advisors: Promoting Cancer Screening and Reducing Disparities.  ( Hilaire DM; 2011) | 1 | 0 | 0 | 0 | 0 | 1 | 1 | 3 |
| Considerations for National Public Health Leadership in Advancing Sexual Health.  (Ivankovich MB, et al.; 2013) | 0 | 0 | 0 | 0 | 0 | 1 | 0 | 1 |
| Chiropractic Care and Public Health: Answering Difficult Questions About Safety, Care Through the Lifespan, and Community Action.  (Johnson, C, et al.., 2012) | 2 | 2 | 0 | 0 | 0 | 1 | 1 | 6 |
| The Social Ecological Model as a Framework for Determinants of 2009 H1N1 Influenza Vaccine Uptake in the United States  (Kumar S, et al.; 2012) | 2 | 2 | 2 | 2 | 1 | 1 | 1 | 11 |
| Issue Editor's Preface: Linkages to Leverage Improvement in Health.  (Larson CO, 2004) | 0 | 0 | 0 | 0 | 0 | 1 | 0 | 1 |
| Public Education and Targeted Outreach to Underserved Women Through the National Breast and Cervical Cancer Early Detection Program.  (Levano W, et al.; 2014) | 0 | 0 | 0 | 2 | 0 | 1 | 0 | 3 |
| Theoretical Approaches to Motivating Change: A Farm Family Case Example  (Lund CH, et al.; 2005) | 0 | 0 | 0 | 2 | 0 | 1 | 0 | 3 |
| Strategies for Increasing Cervical Cancer Screening Amongst First Nations Communities in Northwest Ontario, Canada.  (Maar M, et al.; 2014) | 1 | 2 | 2 | 0 | 1 | 1 | 0 | 7 |
| Prevalence and Correlates of Breast and Cervical Cancer Screening among a Midwest Community Sample of Low-Acculturated Latinas  (Martínez -Donate AP, et al.; 2013) | 1 | 2 | 2 | 2 | 1 | 1 | 2 | 11 |
| Factors Influencing Frontline Health Service Providersʼ Likelihood to Recommend a Future, Preventive HIV Vaccine to Key Populations in Karnataka, South India  (McClarty LM, et al.; 2015) | 1 | 2 | 2 | 2 | 1 | 1 | 2 | 11 |
| Barriers and Facilitators to Testing, Treatment Entry, and Engagement in Care by HIV-positive Women of Color.  (Messer LC, et al.; 2013) | 1 | 2 | 2 | 2 | 1 | 1 | 2 | 11 |
| Social Determinants Associated with Colorectal Cancer Screening in an Urban Community Sample of African-American Men  (Mitchell JA, et al.; 2013) | 1 | 2 | 2 | 2 | 1 | 1 | 2 | 11 |
| Mammography Facilities are Accessible, so Why is Utilization so Low?  (Mobley LR et al., 2009) | 2 | 1 | 0 | 2 | 1 | 1 | 2 | 9 |
| The Role of Religious Values in Decisions about Genetics and the Public's Health  (Modell SM, et al.; 2014) | 1 | 2 | 0 | 0 | 0 | 1 | 0 | 4 |
| A Community-based Participatory Research Approach to Understanding Pap Testing Adherence among Vietnamese American Immigrants.  (Nguyen-Truong CKY, et al.; 2012) | 1 | 2 | 2 | 2 | 1 | 1 | 2 | 11 |
| Applying the Social Ecological Model to Evaluate a Demonstration Colorectal Cancer Screening Program in Louisiana  (Nuss HJ, et al.; 2012) | 0 | 2 | 2 | 2 | 1 | 1 | 1 | 9 |
| PCNA 19th Annual Symposium: Poster Abstracts.  (PCNA, 2013) | 1 | 2 | 2 | 2 | 0 | 1 | 0 | 8 |
| Public Policy Approaches to the Prevention of Heart Disease and Stroke.  (Pearson AT, 2011) | 0 | 0 | 0 | 0 | 0 | 1 | 0 | 1 |
| Linkages Between Clinical Practices and Community Organizations for Prevention: A Literature Review and Environmental Scan.  (Porterfield SD, et al., 2012) | 2 | 2 | 2 | 2 | 0 | 1 | 2 | 11 |
| Social Work in the Emergency Department-implementation of a Domestic and Family Violence Screening Program  (Power C, et al.; 2011) | 2 | 2 | 1 | 1 | 1 | 1 | 0 | 8 |
| Determinants of Mammography in Women with Intellectual Disabilities  (Wilkinson JE, et al.; 2011) | 1 | 2 | 0 | 2 | 1 | 1 | 2 | 8 |
| Social Ecological Predictors of Prostate-specific Antigen Blood Test and Digital Rectal Examination in Black American Men  (Woods VD, et al.; 2006) | 1 | 2 | 2 | 2 | 1 | 1 | 2 | 11 |
| A Community-based Approach to Translational Research Addressing Breast Cancer Disparities.  (Yeary K, et al.; 2011) | 1 | 2 | 2 | 2 | 1 | 1 | 0 | 9 |

**Table S5: Quality assessment of studies using the TTI**

| **Study**  **(author(s), year)** | **Clarity of CRQ(s)/ hypothesis** | **Clarity of data collection methods** | **Clarity of sampling plan** | **Clarity of sampling size** | **Clarity of analysis method** | **Clarity of conclusions** | **Clarity of limitations** | **Overall quality of study** |
| --- | --- | --- | --- | --- | --- | --- | --- | --- |
| Design of a School-based Randomized Trial to Reduce Smoking among 13 to 15-year olds, the X:IT Study.  (Andersen A, et al.; 2014) | 1 | 2 | 1 | 1 | 1 | 1 | 1 | 8 |
| Motivational and Social Cognitive Predictors of Doping Intentions in Elite Sports: An Integrated Approach.  (Barkoukis V, et al.; 2013) | 2 | 2 | 0 | 2 | 1 | 1 | 2 | 10 |
| Illicit Use of Prescription Stimulants in a College Student Sample: A Theory-Guided Analysis  (Bavarian N, et al.; 2013) | 1 | 2 | 2 | 2 | 1 | 1 | 2 | 11 |
| An Exploratory Multilevel Analysis of Nonprescription Stimulant Use in a Sample of College Students  (Bavarian N, et al.; 2014) | 2 | 2 | 2 | 2 | 1 | 1 | 2 | 12 |
| Using Structural Equation Modeling to Understand Prescription Stimulant Misuse: A Test of the Theory of Triadic Influence  (Bavarian N, et al.; 2014) | 2 | 2 | 2 | 2 | 1 | 1 | 2 | 12 |
| A Commentary on the Triadic Theory of Influence as a Guide for Adapting HIV Prevention Programs for New Contexts and Populations: The CHAMP-South Africa Story.  (Bell CC, et al.; 2007) | 0 | 0 | 2 | 2 | 1 | 1 | 0 | 6 |
| The Critical Role of Nurturing Environments for Promoting Human Well-being.  (Biglan A, et al.; 2012) | 0 | 0 | 0 | 0 | 0 | 1 | 0 | 1 |
| Theories of How the School Environment Impacts on Student Health: Systematic Review and Synthesis.  (Bonell CP, et al.; 2013) | 2 | 2 | 2 | 2 | 0 | 1 | 2 | 11 |
| Family First: The Development of an Evidence-based Family Intervention for Increasing Participation in Psychiatric Clinical Care and Research in Depressed African American Adolescents  (Breland-Noble AM, et al; 2006) | 0 | 1 | 0 | 0 | 0 | 1 | 1 | 3 |
| Psychological and Social Risk Factors in Adolescent Smoking Transitions: A Population-based Longitudinal Study.  (Bricker JB, et al.; 2009) | 2 | 2 | 1 | 1 | 1 | 1 | 2 | 10 |
| Determinants of Tobacco Use among Korean Female Adolescents: Longitudinal Test of the Theory of Triadic Influence  (Chun J; 2014) | 1 | 2 | 2 | 2 | 1 | 1 | 2 | 11 |
| Gender Differences in Factors Influencing Smoking, Drinking, and Their Co-occurrence among Adolescents in South Korea.  (Chun J & Chung IJ; 2013) | 1 | 2 | 2 | 2 | 1 | 1 | 2 | 11 |
| The Grady Nia Project: A Culturally Competent Intervention for Low-income, Abused, and Suicidal African American Women  (Davis SP; 2009) | 0 | 0 | 0 | 0 | 0 | 1 | 0 | 1 |
| Determinants of Adolescent Bicycle Use for Transportation and Snacking Behavior  (de Bruijn G, et al.; 2005) | 2 | 2 | 0 | 2 | 1 | 1 | 2 | 10 |
| Predictors of Binge Drinking in Adolescents: Ultimate and Distal Factors - a Representative Study  (Donath C, et al.; 2012) | 1 | 2 | 2 | 2 | 1 | 1 | 2 | 11 |
| Targets for Primary Prevention: Cultural, Social and Intrapersonal Factors Associated with Co-occurring Health-related Behaviors.  (Dusseldorp E, et al.; 2014) | 2 | 2 | 2 | 2 | 1 | 1 | 2 | 12 |
| Effects of 2 Prevention Programs on High-risk Behaviors among African American Youth: A Randomized Trial.  (Flay BR, et al.; 2004) | 2 | 2 | 2 | 2 | 1 | 1 | 2 | 12 |
| A Culturally-informed Approach to Trauma, Suicidal Behavior, and Overt Aggression in African American Adolescents  (Graves KN, et al.; 2010) | 1 | 2 | 0 | 0 | 0 | 1 | 0 | 4 |
| Influences Affecting Adolescent Smoking Behavior in China.  (Grenard JL, et al.; 2006) | 2 | 2 | 2 | 1 | 1 | 1 | 2 | 11 |
| Concurrent Predictors of Cigarette and Alcohol Use among U.S. and Russian Adolescents  (Gunning M, et al.; 2009) | 2 | 2 | 0 | 2 | 1 | 1 | 2 | 10 |
| A Multilevel-based Study of School Policy for Tobacco Control in Relation to Cigarette Smoking among Children in Elementary Schools: Gender Differences.  (Huang HL, et al.; 2010) | 1 | 2 | 2 | 1 | 1 | 1 | 2 | 10 |
| School-level Contextual Factors Associated with Betel Quid Chewing among Schoolchildren in Taiwan.  (Huang HL, et al.; 2009) | 1 | 2 | 2 | 1 | 1 | 1 | 2 | 10 |
| Starting to Smoke: A Qualitative Study of the Experiences of Australian Indigenous Youth.  (Johnston V, et al.; 2012) | 1 | 2 | 2 | 2 | 1 | 1 | 2 | 11 |
| Suicidal, Abused African American Women's Response to a Culturally Informed Intervention  (Kaslow NJ, et al.; 2010) | 2 | 2 | 2 | 2 | 1 | 1 | 2 | 12 |
| Cultural, Social and Intrapersonal Factors Associated with Clusters of Co-occurring Health-related Behaviors among Adolescents.  (Klein Velderman M, et al.; 2015) | 1 | 2 | 0 | 2 | 1 | 1 | 2 | 9 |
| Brief Report: The Adaptation of Project Northland for Urban Youth.  (Komro KA, et al.; 2004) | 0 | 2 | 0 | 1 | 0 | 1 | 0 | 4 |
| Participation in a Sigmoidoscopic Colorectal Cancer Screening Program: A Pilot Study  (Kremers SP, et al.; 2000) | 1 | 2 | 2 | 2 | 1 | 1 | 2 | 11 |
| Description of an Efficacious Behavioral Peer-driven Intervention to Reduce Racial/Ethnic Disparities in AIDS Clinical Trials.  (Leonard NR, et al.; 2013) | 2 | 0 | 0 | 0 | 0 | 1 | 2 | 5 |
| Health-promoting and Health-risk Behaviors: Theory-driven Analyses of Multiple Health Behavior Change in Three International Samples  (Lippke S, et al.; 2012) | 2 | 2 | 2 | 2 | 1 | 1 | 2 | 12 |
| Predicting Self-initiated Marijuana Use Cessation among Youth at Continuation High Schools.  (Little MA, et al.; 2013) | 2 | 2 | 2 | 2 | 1 | 1 | 2 | 12 |
| The Development and Implementation of Theory-driven Programs Capable of Addressing Poverty-impacted Children's Health, Mental Health, and Prevention Needs: CHAMP and CHAMP+, Evidence-informed, Family-based Interventions to Address HIV Risk and Care.  (McKay MM, et al.; 2014) | 0 | 0 | 0 | 0 | 0 | 1 | 2 | 3 |
| A Multilevel Analysis Examining the Association Between School-based Smoking Policies, Prevention Programs and Youth Smoking Behavior: Evaluating a Provincial Tobacco Control Strategy.  (Murnaghan DA, et al.; 2008) | 2 | 2 | 0 | 2 | 1 | 1 | 2 | 10 |
| Cultural and Social Influences on Food Consumption in Dutch Residents of Turkish and Moroccan Origin: A Qualitative Study.  (Nicolaou M, et al.; 2009) | 1 | 2 | 2 | 2 | 1 | 1 | 2 | 11 |
| Adolescent Psychological and Social Predictors of Young Adult Smoking Acquisition and Cessation: A 10-Year Longitudinal Study.  (Otten R, et al.; 2011) | 1 | 2 | 0 | 2 | 1 | 1 | 2 | 9 |
| Project Northland: Long-term Outcomes of Community Action to Reduce Adolescent Alcohol Use.  (Perry CL, et al.; 2002) | 2 | 2 | 0 | 2 | 1 | 1 | 2 | 10 |
| A Review of Similarities between Domain-specific Determinants of Four Health Behaviors among Adolescents.  (Peters LWH, et al.; 2009) | 2 | 2 | 2 | 2 | 1 | 1 | 2 | 12 |
| Effects of Transfer-oriented Curriculum on Multiple Behaviors in the Netherlands.  (Peters LWH, et al.; 2015) | 2 | 2 | 2 | 0 | 1 | 1 | 2 | 10 |
| Sexual Violence and Youth in South Africa: The Need for Community-based Prevention Interventions  (Petersen I, et al.; 2005) | 1 | 2 | 2 | 2 | 1 | 1 | 0 | 9 |
| Youth Culture and Smoking: Integrating Social Group Processes and Individual Cognitive Processes in a Model of Health-Related Behaviors.  (Schofield PE, et al.; 2003) | 2 | 2 | 2 | 2 | 1 | 1 | 2 | 12 |
| Maternal Expectations, Mother-Child Connectedness, and Adolescent Sexual Debut.  (Sieving RE, et al.; 2000) | 2 | 2 | 0 | 1 | 1 | 1 | 2 | 9 |
| Community Perceptions of Adequate Levels and Reasons for Skin Protection.  (Stanton WR, et al.; 2005) | 1 | 2 | 2 | 2 | 1 | 1 | 2 | 11 |
| The One-year Prospective Prediction of Substance Abuse and Dependence among High-risk Adolescents.  (Sussman S, et al.; 2000) | 1 | 2 | 0 | 2 | 1 | 1 | 2 | 9 |
| Concurrent Predictors of Drug Use Consequences among U.S. and Russian Adolescents.  (Sussman S, et al.; 2009) | 2 | 2 | 0 | 2 | 1 | 1 | 2 | 10 |
| Prospective Predictors of Technology-Based Sexual Coercion by College Males.  (Thompson MP & Morrison DJ; 2013) | 2 | 2 | 2 | 2 | 1 | 1 | 2 | 12 |
| Obesity Prevention in Low Socioeconomic Status Urban African-American Adolescents: Study Design and Preliminary Findings of the Health-Kids Study.  (Wang Y, et al.; 2006) | 1 | 2 | 2 | 1 | 1 | 1 | 0 | 8 |
| Developmental Trajectories of Cigarette Use and Associations with Multilayered Risk Factors among Chinese Adolescents.  (Xie B, et al.; 2013) | 1 | 2 | 2 | 2 | 1 | 1 | 0 | 9 |
